# A Review of Simulation Tools Utilization for the Process of Laser Powder Bed Fusion

**DOI:** 10.3390/ma18040895

**Published:** 2025-02-18

**Authors:** Ľuboš Kaščák, Ján Varga, Jana Bidulská, Róbert Bidulský, Tibor Kvačkaj

**Affiliations:** 1 Department of Technology, Materials and Computer Supported Production, Faculty of Mechanical Engineering, Technical University of Košice, Letná 9, 04002 Košice, Slovakia; lubos.kascak@tuke.sk (Ľ.K.); jan.varga@tuke.sk (J.V.); 2 Institute of Materials, Faculty of Materials, Metallurgy and Recycling, Technical University of Košice, Letná 9, 04200 Košice, Slovakia; 3 Bodva Industry and Innovation Cluster, Budulov 174, 04501 Moldava and Bodvou, Slovakia; director@biic.sk (R.B.); tibor.kvackaj@biic.sk (T.K.); 4 Advanced Research and Innovation Hub, Budulov 174, 04501 Moldava and Bodvou, Slovakia

**Keywords:** simulation process, metal additive production, support materials, distortion

## Abstract

This review describes the process of metal additive manufacturing and focuses on the possibility of correlated input parameters that are important for this process. The correlation of individual parameters in the metal additive manufacturing process is considered using simulation tools that allow the prediction of various defects, thus making the real production process more efficient, especially in terms of time and costs. Special attention is paid to multiple applications using these simulation tools as an initial analysis to determine the material’s behavior when defining various input factors, including the results obtained. Based on this, further procedures were implemented, including real production parts. This review also points out the range of possible variations that simulation tools have, which helps to effectively predict material defects and determine the volume of consumed material, supports construction risk, and other information necessary to obtain a quality part in the production process. From the overview of the application of simulation tools in this process, it was found that the correlation between theoretical knowledge and the definition of individual process parameters and other variables are related and are of fundamental importance for achieving the final part with the required properties. In terms of some specific findings, it can be noted that simulation tools identify adverse phenomena occurring in the production processes and allow manufacturers to test the validity of the proposed conceptual and model solutions without making actual changes in the production system, and they have the measurable impact on the design and production of quality parts.

## 1. Introduction

Complex parts are increasingly becoming part of production in important industry areas such as automotive, aerospace, or biomedicine [1,2,3,4,5,6,7,8,9,10,11,12]. The manufactured parts must withstand extreme temperatures and meet all important requirements for functionality and properties. Using the process Laser Powder Bed Fusion known (L-PBF), these demanding requirements can be met, which is why this process is becoming increasingly attractive as a solution for various types of components [13,14,15,16,17,18]. Among other things, the L-PBF process allows parts to be produced economically within the framework of topological optimization [19,20,21]. Therefore, additive manufacturing processes constantly require innovation, especially in the design of various types of components that cannot be produced by conventional methods [22,23,24,25,26]. In this case, we are talking about powder metallurgy technology, where common metal materials can be used to produce parts [27,28]. Currently, the application of metal additive manufacturing is constantly advancing compared to conventional manufacturing methods. The main advantage of this method is the reduction in the material used, as well as energy consumption and production time [29,30,31]. Using modern powder consolidation technology and advanced analytical tools opens the possibility of investigating the mechanisms of material formation during the sintering process under various conditions [32]. Therefore, the application of powder metallurgy is suitable for producing materials with graded properties and, at the same time, offers a transformative approach to the design and processing of materials. As a result, it is possible to produce geometries of complex shapes, print multiple materials at once, achieve the required dimensional accuracy or higher resolution, and at the same time optimize the usability of the material [33,34,35,36].

### 1.1. Process L-PBF

The production of parts using 3D printing, where material is deposited layer by layer, is called additive manufacturing [37,38,39,40,41,42,43,44,45]. However, when using metals, a laser or electron beam is used in this process, which allows for selective melting of the metal powder [46,47,48,49]. In the case of a laser beam, individual layers of metal powder are repeatedly melted and solidified to create parts with almost full density without additional processing steps [50,51].

**Figure 1:**
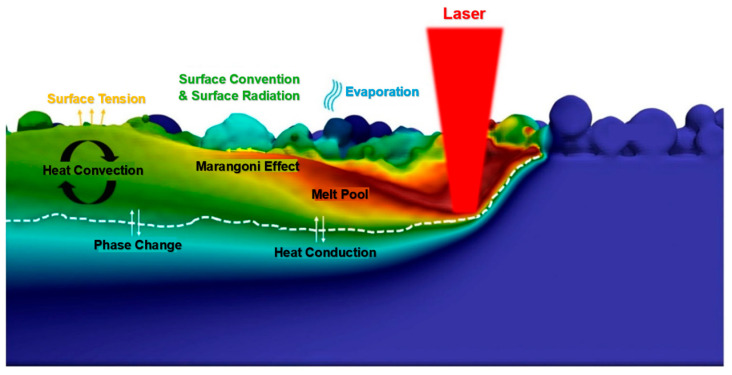
The dynamics of melt pool formation in the L-PBF process [54].

Metal additive manufacturing allows for more efficient production, shorter production times, and thus lower costs [52]. The increasing development and research in metal additive manufacturing are currently bringing a growing demand for using the L-PBF process. Using a laser beam in the part manufacturing process and its high intensity applied to melting metal materials is expected to locally increase the temperature distribution to 1000 °C [53]. Figure 1 shows the dynamics of the formation of a melt pool in the L-PBF process, where factors such as the laser heat source, Marangoni force, surface tension force, rebound force, surface heat, evaporation heat, and conventional losses are created within the interface between the melt pool and the gas. It is also necessary to consider heat conduction and its convention within the melt pool [54].

In the process of creating a melting pool, we can encounter several phenomena that affect the temperature distribution in this pool. Due to the influence of the laser beam, metal powder is heated and melted, resulting in various thermal and physical processes. The action of the laser creates a highly dynamic and fluctuating melt pool, which is accompanied by wetting, recoil, as well as gravitational and capillary forces. In the case of using a low laser power, together with a large layer thickness and a high laser speed, insufficient energy can be generated, which can lead to high surface tension, poor wettability of the molten pool, or non-melting of the powder. Capillary forces tend to drive the melt pool in hydrodynamic motion due to the high surface tension of the molten pool liquid around the edge and low viscosity. The reason for this is the constantly changing shape and size of the melt during the L-PBF process. As a result of the formation of strong temperature gradients arising in the melt pool, a complex hydrodynamic flow is created, which can lead to the formation of spatter, and thus the high-speed surface liquid can spill onto the surrounding powder layer. This is accounted for by the effects of surface tension by strong surface convection flow (Marangoni convection effect) in the transition region. This is an effect indicating the convective flow of molten metal inside the pool driven by the surface tension gradient. A higher Marangoni number results in a larger pool with a high aspect ratio, which in turn helps ensure a proper interlayer and thus low porosity. In addition, the recoil force initiated by the laser input-induced metal vapors creates an additional driving force for the melt flow. The energy density is then high enough to vaporize the metallic elements and form a gas plasma. At the same time, during the L-PBF process, heating and cooling around the transition temperature of the β phase leads to a complete renucleation of the phases—phase change. The melting pool is thus formed from the deposition zone (DZ) at the top, the remelting zone (RZ) at the bottom, and the heat-affected zone (HAZ) at the bottom [55,56,57].

Metal additive manufacturing enables the production of structurally reliable parts, which ultimately requires understanding the entire process, its underlying physical processes, the materials used, individual process control methods, and predicting future defects. In producing these complex shapes, the need for material removal, e.g., by drilling and milling operations, is minimized [58,59,60,61]. Among other advantages mentioned in the metal additive manufacturing process is waste-free production due to the recycling of most unmelted powder [62].

### 1.2. Possibilities of Predicting Negative Impacts in the L-PBF Process

The high geometric complexity achieved using metal additive manufacturing is relevant [63,64]. However, short-time interactions result in large thermal gradients, which result in thermal stresses. The occurrence of large thermal gradients, as well as the rapid solidification process of the part in the L-PBF process, can cause not only high residual stresses, but also excessive thermal distortion, which results in part failure or worse mechanical properties of the part. One of the methods to reduce the temperature gradient in the metal additive manufacturing process is the preheating method, which can effectively inhibit the formation and accumulation of residual stress. Likewise, preheating the motherboard will reduce thermal gradients and reduce stress. Another option would be the use of thermocouples, allowing direct measurement of heating/cooling curves and subsequent thermal velocities and gradients according to the nature of sample production in the LPBF method. However, it is necessary to consider that they are often located relatively far from the laser traces, which makes it difficult to collect information about the thermal development in the process zone. The use of high-speed infrared cameras to measure temperatures in the corresponding ranges of radiation wavelengths is also considered. Other measurement options would include the use of X-ray Diffraction (XRD), which is used to measure changes in the atomic spacing of a material during the L-PBF process and correlate them with temperature evolution based on the material’s thermal expansion [65,66,67].

Finally, it is necessary to know the thermal history resulting from the metal printing process. The thermal history, as well as the melting/solidification conditions, influence the subsequent microstructure (thermal ratios), thermal deformation (thermal gradients), and thermal stress precisely with the support of simulation tools. Therefore, the results of calculations from numerical simulation could be used to predict not only the local microstructure, but also thermal deformations.

On the other hand, rapid solidification is manifested by the formation of segregation and the development of non-equilibrium phases [68]. The main research challenges encountered in the L-PBF process include residual stresses and deformations of the part [69,70,71,72,73,74]. Residual stresses tend to deform the parts, depending on the direction of the residual stresses [75]. If residual tensile stresses are present, this is undesirable because they negatively affect the mechanical properties. It can also lead to deformation of the structural geometry and the induction of cracking or delamination. In most cases, tensile residual stresses are generated towards the sample surfaces and compressive ones in the center of the part [76,77,78,79]. Residual stresses are influenced by many factors, such as the heat source and ambient conditions, as well as input process parameters, such as scanning speed, laser power, strategy, etc. Process conditions, material properties, geometry of the part, and types of supports used can affect the amount of residual stress developed in the part. The residual stress itself is caused by two mechanisms that arise in the L-PBF process: the thermal gradient mechanism (TGM) and the cooling phase of the molten top layers. In the case of the TGM mechanism, these are large temperature gradients occurring near the laser spot. The second case occurs during exposure, where the expansion of the heated top layer is limited by the underlying substrate and elastic compressive deformations are created. If the yield point of the material is exceeded, the top layer is plastically compressed [80,81,82,83].

For the assessment of negative impacts, such as residual stresses and deformations of parts, simulation tools use the inherent deformation method for their prediction. As part of it, they apply a calibration step to capture the inherent stress developed for the combination of machine, scanning process, and material. The resulting deformations can be caused by thermal stress in the part, which led to partial delamination of the part from its supports during building. This can result in the deposition of new layers, subsequently creating new layers, which led to the remelting of previous layers, causing them to expand. In order to subsequently avoid these undesirable effects, the manufactured parts are further subjected to the process of hot isostatic pressing (HIP), or heat treatment, which relieves residual stress. Porosity will also be reduced, which will improve the functionality of the part [84].

The correct setting of the laser speed and power in the L-PBF process significantly affects the thermal gradient G (K/m) and the liquid–solid interface velocity in (m/s) of the molten pool. These latter parameters control the solidification process and can be regulated to induce epitaxial grain growth or equiaxed grain growth (grains are randomly oriented and have a size comparable to the layer thickness) [85]. In the L-PBF process, an important parameter is the so-called hatch spacing, the correct selection of which allows the avoidance of linear hollow structures associated with powder denudation effects [86].

**Figure 2:**
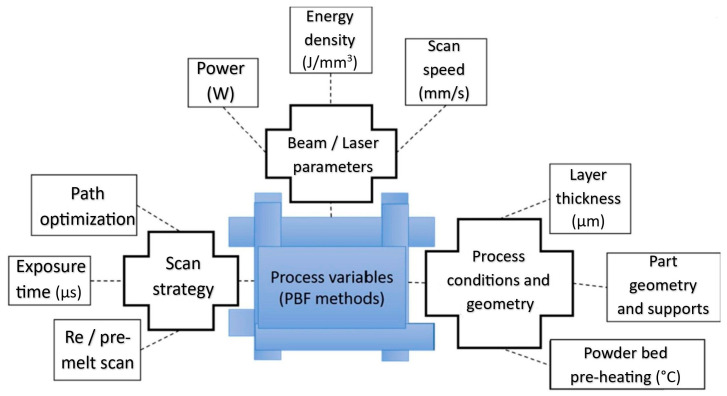
Process parameters in the L-PBF method controlling residual stress characteristics [87].

The variables considering the influence of process parameters on residual stress are shown in Figure 2.

The L-PBF process involves several variables that need to be considered, such as scanning speed, laser power, layer thickness, deposition strategy, etc. The choice of deposition path strategy (zigzag, raster, continuous, contour) affects the development of residual stress and deformation in the part. Parallel and contour deposition strategies show lower effective stress than spiral strategies [88,89]. It is necessary to consider the large temperature gradient that occurs during the metal printing process and, therefore, the complex heat transfer caused by the cyclic processing of the laser beam [90,91]. The combination of these steep temperature gradients and the high cooling rate creates fine columnar grains oriented along the direction of the build. For this reason, the manufactured parts have reduced ductility, increased strength, and increased microstructural and mechanical properties anisotropy, depending on the alloy systems [92,93,94,95]. An advantage of a progressive technology such as metal additive manufacturing is the possibility of recycling the used powder and increasing its % utilization to 100% [96,97].

### 1.3. Importance, Impact and Simulation of Metal Powders in the L-PBF Process

Sustainability in the use of metal powders does not only refer to the use of secondary raw materials for the production of the powder but must also include the recycling and reuse of powders that have undergone the additive manufacturing process. It is this sustainability that can become a direct contributor to the circular economy. Likewise, one of the main industrial challenges of the L-PBF process is not only high thermal gradients, causing deformations and residual stresses, but also the need for sophisticated powder handling and recycling to maintain powder quality. The high-quality powder makes it possible to ensure not only optimal fluidity, which increases the quality of the metal production process, but also the mechanical properties of the final part. Among other things, the quality of the powder is influenced by several factors, including the shape and size of the particles, roughness, density, the presence of impurities, or the chemical composition. Therefore, uniform particle size distribution is necessary to obtain a consistent and smooth powder layer during the manufacturing process. Otherwise, the inconsistent particle size leads to changes in the packing density, which affects the laser absorption in the melting phase as well as the thermal conductivity. As a result, defects such as sphericity, the essence of which is that the unmelted powder forms spherical particles, or insufficient fusion between the layers may occur. The subsequent presence of defects in powders can affect internal defects, surface roughness, mechanical properties, microstructure, and corrosion resistance. Therefore, an option to achieve the reuse of powders is the plasma spheroidization method, in which non-regular particles are transformed into spherical ones with the help of thermal plasma. While creating a unique microstructure, the L-PBF process can also induce metallurgical defects such as pores, inclusions, cracks, and therefore, poor fusion between layers due to improper process control, which can further affect the mechanical properties of the manufactured parts [98,99,100]. Cracks appear if the residual stress value exceeds the material’s tensile strength [101].

In the case of emerging pores, zones of high stress concentration can be formed, which promotes the initiation and propagation of cracks. The mechanical strength can be significantly reduced by the occurrence of irregular or grouped pores due to the reduction in the supporting cross-sectional area. Due to the complex production parameters of the L-PBF process, the main cause of the above-mentioned defects is the instability of the melt, which is significantly affected by rapid heating and rapid cooling. Therefore, the state of the melt has a great influence on the solidification of the molten powder and the consolidation of the layers affecting the final quality of the manufactured part. Defects in parts produced using the L-PBF method are manifested by the formation of porosity due to rapid cooling solidification and complex flow of the molten pool during production, which affects the mechanical properties of the parts produced. They can be divided into gas pores and lack of fusion (LOF) defects according to their formation mechanism. In the case of gas pores, it is possible to distinguish keyhole pores, powder raw material pores, and hydrogen pores. The formation of pore defects, especially keyhole pores, occurs on a spatial scale of microns and a time scale of microseconds. If we focus more on the mechanical properties of the parts, then the tensile properties of the parts are not significantly affected by porosity but are strongly affected by internal LOF defects. In the case of fatigue properties, their influence is based on surface roughness, residual stresses, microstructure anisotropy, and internal pore defects. On the contrary, fatigue cracks form in small notches on the surface in the case when the rough surface is subjected to a dynamic load. It is possible to reduce the porosity and its characteristics by controlling the process, which can change the phase of the material as well as the microstructure [102,103,104].

In case of cracks, the regular and spherical shape of the powder particle is also important. Many alloys tend to solidify in the form of dendritic columnar growth, which causes cracks during solidification. Appropriate nanoparticulate powder materials can promote heterogeneous nucleation, induce fine uniform grain growth, reduce solidification effects, and eliminate hot cracking. Last but not least, the formation of defects in the form of the growth of inclusions mainly occurs in a group of alloys such as stainless steels, where oxide inclusions can form due to the high level of oxygen in the powder. The creation of some typical inclusions, such as MnS, may be reduced due to rapid cooling. The formation of these defects in the form of inclusions can affect not only the mechanical properties and corrosion resistance of stainless steel but also its microstructure. When the L-PBF process is applied to produce various complex or simple parts, some extremely fast interactions may occur between a concentrated laser source and metal powders of a few μm. This results in so-called gas, plasma atomization, or melt centrifugation, but with the difference that instead of powders, a final 3D component is created [105,106].

**Figure 3:**
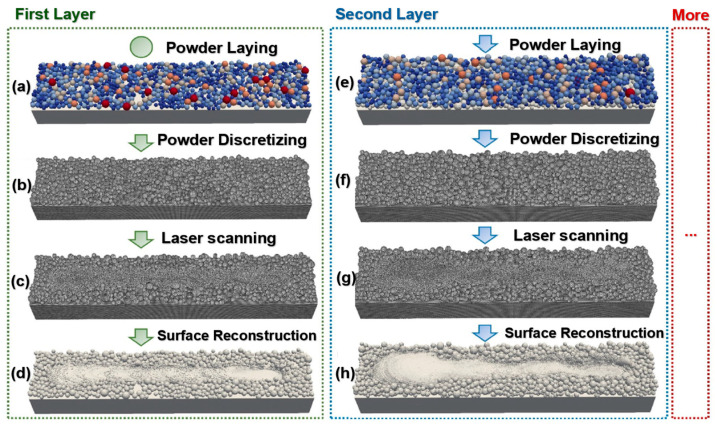
Display of the multi-layer process simulation in steps [54]. (**a**) powder laying on the 1st layer (**b**) powder discretizing on the 1st layer (**c**) laser scanning on the 1st layer (**d**) surface reconstruction on the 1st layer (**e**) powder laying on the 2nd layer (**f**) powder discretizing on the 2nd layer (**g**) laser scanning on the 2nd layer (**h**) surface reconstruction on the 2nd layer.

It is essential to correlate the size and shape of the starting powders with the main process parameters, such as laser power, scan speed, layer thickness, hatching space, and the scanning strategy used to concern the correct manufacturing step [107,108,109]. Although the solidification process and thermomechanics of metal additive manufacturing are very complex in detail, the effects above leading to deformation can be simulated at the macroscopic level. A simulation view of the L-PBF multilayer process, which features alternating powder deposition and laser scanning of each layer, is shown in Figure 3.

In the simulation of the multilayer L-PBF process, a simulation of powder bed generation is performed for each layer using the Discrete Element Method (DEM), as shown in Figure 3a,e. Subsequently, these elements, particles, are divided into particles using the Smoothed Particle Hydrodynamics (SPH) method, as shown in Figure 3b,f. Then, the laser scanning procedure is implemented, as shown in Figure 3c,g.

The second layer powder deposition requires the extracted surface of the first layer powder bed, which is imported as the lower boundary in subsequent simulations (DEM), as shown in Figure 3d,h [54]. Monitoring the residual stress distribution in the part, which depends on its geometry, is also justified. The stress redistribution that occurs when the part is removed from the base plate is an interesting phenomenon that simulation can explain and investigate. In areas where the Von Mises stress is equal to the material’s yield strength, plastic deformation can also indicate material damage and the risk of failure. Several materials are used in metal additive manufacturing, and research into their further development is ongoing. Various metallic materials, as well as alloys, stainless steel [110,111,112], aluminum alloys [113,114,115], nickel-based alloys [116], or titanium alloys [117,118], are materials that can be applied in the metal additive manufacturing process. For this reason, metal powders are the basic materials for producing metal parts using powder metallurgy techniques. Since various processes produce metal powders, spherical powders are mainly used for metal additive manufacturing [119]. Each different property of the powder can affect several properties of the construction process and thus can affect different aspects of the quality of the final part [120,121].

The L-PBF method using aluminum alloys is important for industrial purposes, as the finished parts achieve higher strength and hardness than castings. Due to the high fluidity of the liquid phase, cast Al-Si-Mg alloys are often investigated, which thus facilitates the preparation of pore-free samples and shows excellent weldability [122]. The L-PBF method is also among the most used for producing high-strength steels of class 300 [123,124,125,126]. Another suitable alternative is the possibility of creating an assembly of several parts, as well as the possibility of obtaining parts with high density [127,128].

### 1.4. Application of Metal Additive Manufacturing Technology

It is challenging to produce high-quality parts that meet functional requirements, printing costs, and mechanical properties [129]. Therefore, providing information on simulation tools and their development and prediction capabilities can contribute to more effective use and understanding, a key factor in the metal additive manufacturing process [130]. Applying simulation tools in this process allows for improved quality and thus reduces the number of failures in constructing the part if the correct type of application is identified. Although all parts and materials show the same behavior during the simulation process, the strength of the effects varies depending on the material, geometry, and size of the part. The larger the part, the greater the chance of failure and distortion. Therefore, it is advantageous to use simulation before production begins for large structures, mainly due to the high costs associated with creating large structures. While manufacturing problems can be solved by finding the best orientation, optimal support structures, and appropriate design for metal additive manufacturing, distortion problems can sometimes be avoided [131].

Even heat treatment cannot reduce the shrinkage that occurs during the process. A “pre-deformation” method can be used to avoid high deviations on large structures. Based on simulated displacements, the geometry is adjusted so the shrinkage during the process results in an accurate part. This step then becomes another part of the digital job preparation workflow. Components that were previously impossible to realize due to distortion problems can now be easily and accurately printed using a simulation-based workflow. In recent years, the L-PBF and EBM processes for processing Ti-6Al-4V have made significant progress, especially in process optimization and property characterization [132,133]. It is necessary to constantly address the energy cost growth, the unpredictability of errors, and dimensional inaccuracies in the final parts produced by the metal additive manufacturing process. Reproducibility, speed and correct production require a synergistic approach, which can be best achieved using appropriate simulation tools. Given the high demands on the research of the formation of microstructures and changes in mechanical properties caused by microstructural changes, it is necessary to combine existing experimental and theoretical knowledge on the use of simulation tools to explain the required phenomena occurring in metal additive manufacturing and, therefore, evaluate their progress. The main focus of this review is based on the relationship between the input parameters entering the simulation process and the results, which enables a better understanding of them.

Manufacturing products through efficient processes that limit environmental harm while conserving energy and natural resources aligns with the principles of Sustainable Manufacturing. Although numerous studies have explored the technologies and applications of additive manufacturing, relatively few have examined this approach from a sustainability perspective and its environmental impacts. There remains a critical need to investigate various sustainability aspects of additive manufacturing, including its economic, environmental, and social dimensions. This includes exploring circular economic practices, conducting sustainability evaluations and life cycle assessments, identifying opportunities for sustainable implementation, and addressing the challenges involved [134,135].

Applying metal additive manufacturing technology can also be considered environmentally sustainable and produces less CO_2_ emissions in the context of strengthening the circular economy. It also finds its justification in the aerospace industry, where low weight is required for individual structures, which impacts reducing fuel and, thus, CO_2_ emissions. This is because the part’s design minimizes weight while simultaneously fulfilling the functionality with the desired character [136,137,138]. As a result, metal additive manufacturing is a suitable solution for solving challenges in improving the efficiency and effectiveness of production processes [139,140].

Herzog et al. [141], in their paper, focused on describing the complex relationship between individual additive manufacturing processes and the microstructure and resulting properties of metals. They analyzed the individual structural grains formed under the influence of a complex thermal cycle and high cooling rates in more detail. Compared with conventional forming methods, the metal additive manufacturing process involves layer by layer and the consolidation of powder material into various configurations, using CAD-controlled, selective melting of precursor powder beds [142,143,144,145,146,147,148,149,150].

In this mode, metal evaporation controls the depth of the molten pool [151]. This process has a complex, non-equilibrium physical and chemical metallurgical nature that varies depending on the material and method used. Therefore, parameters such as laser power, scanning speed, etc. affect the characteristics of the molten pool [152,153].

High recoil and Marangoni convection occur in the L-PBF process of 316 L stainless steel, significantly impacting the melt paths. This analysis and the various mechanisms of pore formation were addressed in a study by Khairallah et al. [154]. The result was three differentiable regions: a depression region located at the laser spot, a tail region of the melt path near the end, and a transition region between them. The author used a laser power of 200 W, a scanning speed of 1.5 m/s, and a powder thickness of 27 µm as input parameters. Thijs et al. [155] focused on the importance of scanning parameters and scanning strategies and their impact on the microstructure in a metal additive manufacturing process, where a martensitic structure was observed due to rapid cooling.

The input parameters were a powder layer thickness of 30 µm, a laser power of 42 W, a hatching space of 75 µm, and a scanning speed of 200 mm·s^−1^. The analysis demonstrated the formation of elongated grains due to epitaxial growth, with their direction related to the process parameters.

**Figure 4:**
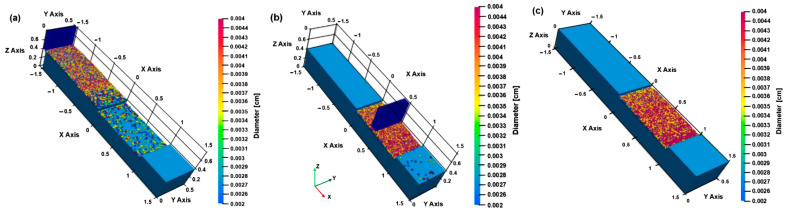
Powder particle size: (**a**) powder settling and powder lying (**b**,**c**) powder spreading and settling to the working zone [158].

At the same time, this correlation of input parameters for the manufacturing process is accompanied by results in the form of application solutions. Based on various studies, integration in the form of a combination of CAD and metal additive manufacturing helps to improve manufacturing and design processes [156]. Therefore, modeling and simulation are important in defining the relationship through process parameters—internal variables, which include solid phase transformation, precipitate formation, or grain morphology in the solidification phase [157]. An example of displaying the size of powder particles in the L-PBF process is shown in Figure 4.

Other authors have assessed the need for CAD tools in the context of topological optimization for metal additive manufacturing processes [159,160,161,162,163,164]. The main technical challenges that prevent the full penetration of additive manufacturing into the industry are the development of knowledge, tools, rules, processes, and methodologies for additive manufacturing [165]. This paper comprehensively reviews several scientific contributions to examine various aspects of integrating simulation tools into the metal additive manufacturing process, not only regarding trends, considerations, techniques, and applications. More understanding and implementation of metal additive manufacturing are needed to prevent designers and engineers from using it to its maximum potential and achieving effective results. Therefore, progress is assessed not only in numerical modeling, but also in applying simulation tools.

For simple parts, the orientation of the part can be determined by the designer’s experience, but in the case of complex parts, integrating simulation tools is very beneficial [166,167].

The discussion regarding simulation tools and their application in the metal additive manufacturing process must cover not only the material characteristics themselves, the definition of the necessary input parameters, and calculation algorithms but also the results in deformations, residual stresses, etc. [168]. Therefore, applying simulation tools within individual manufacturing processes provides results based only on calculation data. However, it does not consider the practical obstacles encountered directly during the real phase of part production. As a result, the verification of simulation results with results in the form of experiments is very important.

This review focuses on understanding the variants enabling the use of simulation tools designed for metal additive manufacturing processes to create an overview of the application of simulation tools, including the obtained results and their application in the real area. This review paper aims to guide researchers in examining the current situation of applying simulation tools in the metal additive manufacturing process and understanding their significance.

The following chapters are aimed first at getting to know the simulation tools that can be applied for the L-PBF process. Their importance is considered not only on a theoretical level, but also in terms of physical phenomena related to powder dynamics, hydrodynamics, etc. The sub-chapter analyzes in more detail different time and spatial scales, scales (microscale, mesoscale and macroscale) which essentially include all the phenomena taking place in the molten bed, as well as their very effect on efficiency, phase transformation, which in turn influences the formation of microstructure, or errors in the L-PBF process. Other subsections describe the most commonly used simulation software (Simufact Additive 2020, 2022, 2023, Ansys Additive 2020R1, Deform, Amphyon, Netfabb Simulation, VGSTUDIO MAX, AscentAM, Altair Inspire Print3D 2020), which can be applied for the simulation process of metal additive manufacturing L-PBF.

Chapter 3 is dedicated to specific applications; the shape and dimensions of the parts are listed in Table 1. Within this review, simple as well as shape-complex parts were selected for various industries, such as automotive, aviation, or engineering industry. This review does not focus only on one type of material used, but offers an analysis of the use of simulation software for different types of materials such as aluminum and titanium alloys, or materials such as stainless steel 316 L, Inconel, etc.

The main importance of applying simulation software for this area is not only predicting various errors but also ensuring the quality of part production. For this reason, further subsections were divided into areas such as thermal phenomena taking place in the process, part orientation and creation of support material, volume fraction, deformation of the part, residual stress, and shape deviation. They can significantly influence the functionality, quality, and dimensional accuracy of the metal part produced by the L-PBF process. Each of these mentioned areas is analyzed in relation to the use of numerical simulation tools in the L-PBF process. As part of the review, the largest possible portfolio of results was analyzed, including more than 300 studies from various authors who dealt with the application of simulation tools in the L-PBF process. The last subsections of this review describe other possibilities that can contribute to the expansion of knowledge about the applicability of simulation tools with regard to future research.

## 2. Simulation Softwares in the Metal Additive Manufacturing Environment

In the case of the L-PBF process, which is very complex, simulations are a tool to predict future errors and understand the correlation of individual parameters and to what extent they are related. There are dynamic changes in the temperature profile, leading to geometric and mechanical deviations in the manufactured parts [133]. The iterations themselves allow for optimizing the process parameters to the final form, which results in a quality part and does not take up much time, as well as material consumption, reducing time and costs [169]. The advantage of applying these simulation tools before the production process is the savings in material, time, and costs [170]. First of all, it is a thermomechanical model that includes the entire thermal history, as well as residual stress. Although process optimization is always necessary, the simulations themselves are a starting point for researchers to produce a part within the framework of an overview of the given process from start to finish. In case of unsatisfactory results, the process can be optimized and subjected to further iteration until it provides the best possible results.

Working with input data in numerical simulation plays an important role, as incorrect results can affect the final shape and properties of the future part, such as porosity, distortion, cracks, deformations, etc. Within the laser–metal interaction, these errors can affect not only the overall microstructure and mechanical properties (fatigue, creep behavior, ductility, strength) but also the density of the part, surface properties, or tribological properties [171,172,173,174,175]. Correctly set process parameters are important for obtaining a part without porosity and thus play an important role in defining the final properties of the part [176,177]. Simulation tools are equally suitable for predicting physical phenomena occurring in the metal additive manufacturing process and thermal-mechanical behavior, including, e.g., heat transfer, temperature distribution, residual stress, deformation, melt pool geometry, phase transformation, or Marangoni convention [178]. These temperature changes significantly determine microstructural changes and final mechanical properties [179,180]. In the final stage, the resulting microstructure significantly differs from the structure obtained by heat treatment, casting, or thermomechanical processing of metals. The reason is the rapid heating rate and subsequent cooling during the metal printing process. This is accompanied by additional complications due to the repeated process of heating and cooling in some areas [181]. Incorrect choice of process parameters can affect errors in the production process and deteriorated properties due to heat and mass transfer, temperature gradient, or non-equilibrium solidification [182]. Much research is being carried out to optimize the L-PBF process using simulation tools. Despite the complexity of the process, which is the L-PBF process, there are always certain gaps in the results [183]. Therefore, various simulation tools are currently being tested to increase the accuracy of the results as much as possible. By using CAD models representing the future shape of the part using a suitable simulation tool that would predict the metal printing process, these computer models can be transformed into physical objects with the desired properties, shape, and dimensions [184]. This process allows us to achieve the shape of any geometry intended for simple or complex parts [185,186,187,188,189]. It is very difficult to predict the material’s behavior in the production process and thus predict the behavior of the selected material [168]. In any production process, the influence of the process parameter settings plays a significant role [183].

### 2.1. CAD Models and Their Importance for the L-PBF Process

The L-PBF process consists of eight main steps, which include the created CAD model, the generated file in *.stl format, the correct orientation of the part, the method of placing the support structure, the construction process, the construction of the part, the removal of the part from the base plate, and post-processing. Therefore, the field of construction, as well as software that allows for the prediction of errors in the manufacturing process, is significant and plays an important role in achieving an efficient manufacturing process [122].

Any error in the initial phase can affect the quality and desired properties of the part. For this reason, attention should be paid not only to the CAD model but also to the choice of part orientation, the correct choice of the support structure, and other input parameters that affect the resulting shape and properties of the part [107,190]. Optimization of the input parameters required in the L-PBF process requires a longer research time [191]. Therefore, one of the alternatives to achieve this optimization is the use of simulation tools. As a result, the time and material costs required for this process are reduced [192] since simulation, even in the case of obtaining non-optimal results, thus replaces the trial and error that would occur in the case of real production. Another iteration in the form of a repeated simulation process with new optimized production conditions can prevent such trial and error and thus achieve effective results, ultimately determining real production practically without errors in the metal printing process [193]. They, therefore, become beneficial not only in the analysis of stress distribution and mechanisms of defect formation but also in the analysis of temperature changes [194]. The essence of applying simulation tools in metal additive manufacturing is to provide a comprehensive connection in the process—structure—properties relationships [195]. Several software programs are currently designed to prepare simulation-based processes for additive metal processing, significantly contributing to the emergence of faster and more efficient engineering processes. If simulation is used to estimate the residual stress, its estimate would be based on the thermal deformations and the temperature difference during the cooling phase. It would also be possible to evaluate these defects with more advanced procedures, such as by X-ray, neutron diffraction, drilling holes, or the bridge curvature method, when the tested part is printed in the shape of a bridge. Information about the residual stresses resulting from a specific combination of additive materials and processes can be obtained by measuring the resulting corrugation [196,197].

### 2.2. Multiphysics Simulations of the L-PBF Process and Their Characteristics

**Figure 5:**
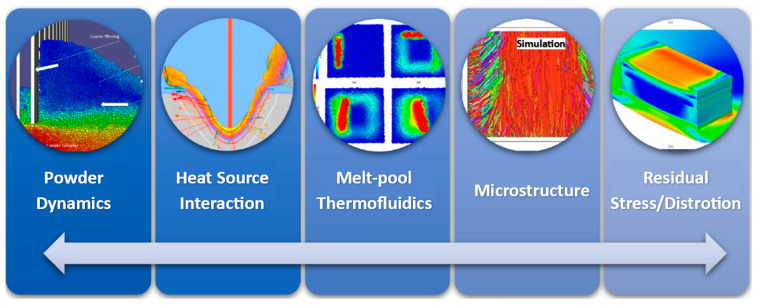
Multiphysics numerical simulations designed for modeling metal additive manufacturing [198].

The microscale enabling the analysis of phenomena related to the melting pool or the structure of emerging grains must, in the case of application of simulation tools, contain functions enabling the analysis of thermodynamic and hydrodynamic effects. Within the computational model of fluid dynamics, it should contain all the phenomena described above in the form of Naivné-Stokes equations, as well as mass and energy conservation equations. In the case of mesoscale, the application of simulation tools for metal additive manufacturing using the Discrete Element Method (DEM) is limited mainly in terms of high computational costs. For this reason, it is difficult to implement these simulation calculations in industry. One possibility could be the use of computers with better specifications, or if another effective principle of amplifying the results of the discrete element method from the mesoscale to the macroscale could be developed. Solving problems in the macroscale is possible using DEM, which is suitable from the point of view of the requirements for studying at the level of individual particles in the LPBF process. It can simulate several discrete particles and represent their physical phenomena. A limitation in this case at the macroscale level may be that it is not possible to describe the plastic deformation and fusion of solid particles. Therefore, one of the possibilities to overcome the mentioned problem is the integration of DEM with other mesoscale numerical approaches. With current models, the discrete element method contributes fidelity within the powder bed geometry only in the initial state of the model. Therefore, it is necessary to keep in mind that the DEM method does not work due to the discrete nature of the powder particles; however, combining this method with the computational fluid dynamics CFD method could solve this problem. In metal additive manufacturing and multiphysics modeling, it recognizes five key elements. Figure 5 shows powder dynamics, heat source interaction melt-pool thermofluidics, microstructure evolution, and stress/distortion [198].

In simulations of mesoscopic additive manufacturing, it is effective to apply the method of smoothed particle hydrodynamics (SPH). During the L-PBF process and due to the influence of the ambient gas present during the process, a finite element (FE) model is developed to observe the dynamics of the powder bed. This makes it possible to simulate the behavior of the melt in the L-PBF process. This simulation makes it possible to simulate all important physical phenomena such as wetting, back pressure, or the Marangi effect, enabling the dynamics of the melting pool to be captured in detail. The action of the laser and its impact transforms atomized metal powder grains into a monolithic part, with the rapid phase transformations that occur here, as well as the high temperature gradients, ultimately defining the shape and quality of the part [198,199].

The geometry initially consists of a substrate covered with a layer of powder grains, and the heat source is a laser impinging on the surface. The generated heat is mostly transferred to the material, but with smaller dominant heat losses through convection and radiation heat transfer. A phase change from solid to liquid and vice versa is required to achieve overall powder fusion modeling, taking into account the latent heat of fusion, with the morphology of the resolidified melt depending on the degree of wetting of the powder particles. Due to low wettability, spherical balls known as the Plateau–Rayleigh instability can be formed, which can be manifested by a significant change in the thickness of the next powder layer, because the previously solidified layer has an irregular and wavy surface. Therefore, the simulation of the melt pool can take into account the heat transfer between the melt pool and the surrounding powder bed and solidified metal [200,201,202].

As a result of the action of the laser during the L-PBF process, gradual absorption of heat can occur, as well as melting of the material or evaporation of the liquid, as well as other complex interactions between the laser and the material. Based on the solidification process of the liquid melt pool, as well as the type of liquid flow, heat transfer profile, and solidification speed, the phase transformation is subsequently determined, which has a significant impact on the formation of the microstructure. On the other hand, another factor that significantly affects the resulting microstructure of the part is the temperature gradient occurring in the melt pool [203,204].

Within the resulting microstructures, the following types can be distinguished: dendrites, planar, cellular, columnar dendrites, or equiaxed grains, while the columnar and cellular dendritic structure occurs in common metal alloys due to a lower temperature gradient and solidification speed. For this reason, the rate of solidification and the temperature gradient have a significant effect on grain growth in the melt pool. Since temperature gradients influence residual stresses and deformations, there are three types of residual stresses. In the first case, it can be a residual stress I known as macro residual stress, and its length scale is larger than the grain size (greater than 1 mm). In the case of residual stress II, it is an intercrystalline residual stress that exists in the range of several grains (0.01–1.0 mm), and in the last case we can encounter residual stress III, which is limited to the range of crystal grains (it is less than 0.01 mm). It can also happen that the high temperature gradients created during the printing process can lead to thermal deformations, which will ultimately affect the mechanical properties of the manufactured parts [205,206].

**Figure 6:**
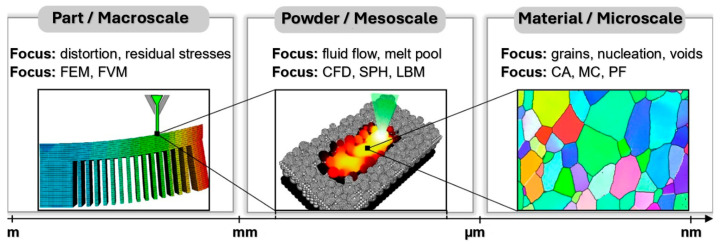
Different representations of scales occurring in the L-PBF process [208].

Most of the phenomena occurring in the L-PBF process occur at different times and spatial scales, as shown in Figure 6. This technology allows parts to be produced with a feature size of 150 μm, which indicates a high resolution. We distinguish three scales: microscale, mesoscale, and macroscale [199,200,201,202].

- The microscale represents phenomena near the laser mass (melting pool generation, the role of interfacial forces in its development and fluid convention). At this scale, denudation, i.e., the formation of defects, can be detected, and the thermal cooling rate, which influences the microstructure, can also be captured. Overall, it includes the phase transformation in the solid state, the grain structure, the direction of their orientation, etc. [203].
- Mesoscale measures melt analysis and stress modeling without considering the effect of phase transition in the solid phase [204]. However, it allows scanning a given layer’s entire layer or regions (scanning pattern). It can also observe factors affecting the local cooling time, such as the scanning pattern’s width, the scanning vector’s length, the flow of molten liquids, and melting and solidification (Marangoni effect) [205].
- The macroscale represents factors such as powder, part geometry (overhangs, element thickness), the influence of the structural plate acting as a heat sink, and conduction through supports. This type of scale also allows tracking defects such as cracks, support separation, residual stress, or deformations [206,207].

**Figure 7:**
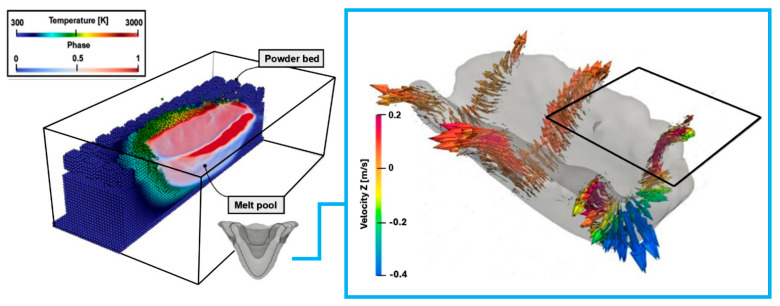
3D simulation of hydrodynamics and the effects of recoil and Marangoni forces [208].

The choice of the required scale also needs to be considered, as physical interactions between the manufacturing process and the formed surfaces can occur at multiple scales during production [209]. At the beginning of the L-PBF process, the hydrodynamics significantly impact the overall process, which takes place throughout the entire build time. Figure 7 shows a fine-resolution representation of the hydrodynamics of the smoothed particles to quantify the effects of recoil and Marangoni forces on the geometry of the melt pool.

**Figure 8:**
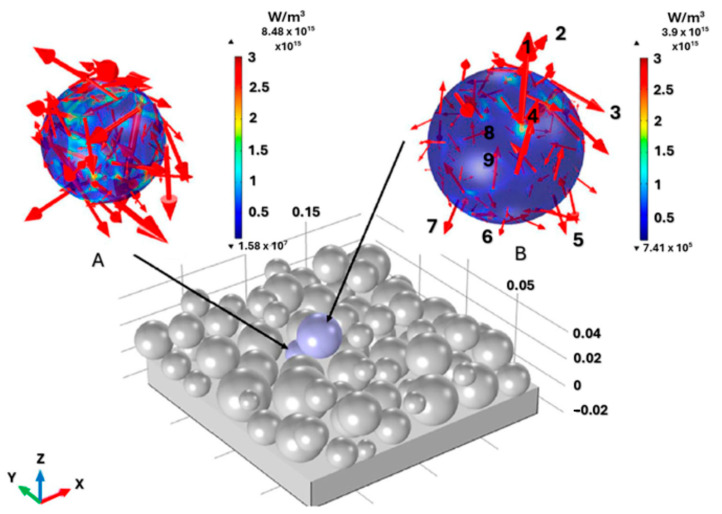
View of laser–powder interaction in the L-PBF process: (**A**) particle with size of 19.2 μm, (**B**) particle with size of 29.2 μm [210].

The heat source and its interaction with the material are closely related to hydrodynamics because they define the thermal boundary condition of the energy balance equation throughout the process. Subsequently, in the L-PBF process, the heat source model describes the beam’s interaction with the powder particles and the resulting thermal profile. Figure 8 [210] approximates the laser–powder interaction in the L-PBF process. Arrows 1–4 represent the reflected part of the laser beam into the environment. Arrows 5–7 indicate multiple reflections of the laser beam through the gap into the powder bed, and arrows 8–9 represent the absorbed energy of the laser beam by the particles.

**Figure 9:**
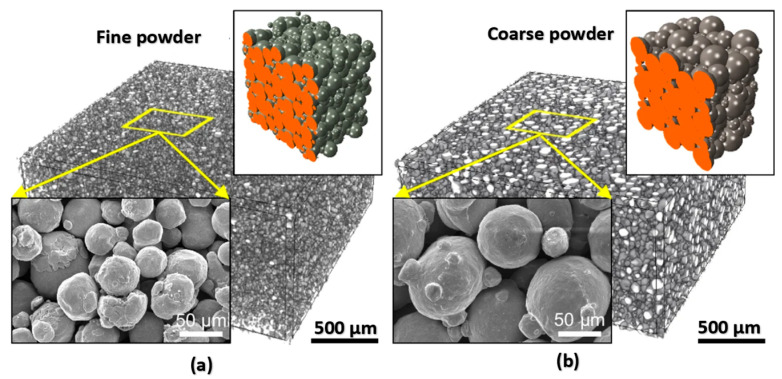
3D rendering of (**a**) fine (**b**) coarse sintered powder bed. Yellow frames show comparison of the magnified area of powder bed of both type of powder [211].

The main result of the interaction of the laser beam with powder particles is the microstructure, the control of which is highly desirable for achieving excellent mechanical properties [211]. The microstructure of parts produced by the L-PBF process differs significantly from that of wrought or cast parts [212,213]. In wrought alloys, the microstructure contains equiaxed grains after proper heat treatment, and the grain size can be considered uniform. However, in alloys produced by the L-PBF process, such as Inconel 625, columnar grain growth usually occurs along the building direction (BD) and can deviate from the building direction [214]. An interesting phenomenon is that due to the increased cooling rate in the L-PBF process, it is possible to achieve a specific microstructure of the material down to the nanoscale [215]. A 3D rendering of the fine and coarse sintered powder bed, which was analyzed using μ-CT scanning, along with their corresponding simulated particle microstructure, is shown in Figure 9 [211]. Representative 3D renderings of fine (Figure 9a) and coarse (Figure 9b) sintered powder beds analyzed by μ-CT scanning along with their corresponding simulated particle microstructure. The insets are FESEM micrographs of fine and coarse sintered powder beds taken at the same magnification.

When the laser beam and secondary parameters in the L-PBF process are applied, residual stress calculation is often encountered, which is realized by simulating the transition temperature in the entire area. Then, the obtained thermal history is used to calculate the development of stress and deformation in thermomechanical simulations. However, the accuracy of the obtained results depends on the quality of the thermal history [216]. The last parameter, in the form of residual stress, represents a challenge due to the large deformation that occurs after the removal of the part from the base plate. Heat-induced deformations in the L-PBF process are estimated to cause almost 70% of failures [217]. In some cases, deformation may not occur during the metal printing process, but when the part is separated from the base plate, residual stresses are released, which deforms the part [218,219,220]. Similarly, the deformation in the L-PBF process results from mass transfer caused by the inhomogeneous temperature distribution [207,221]. Changing the printing process parameters to eliminate or minimize distortion requires consideration of many parameters, as well as considering the influence of different metal powders and understanding the behavior of specific materials under thermal loading. Therefore, simulation of the metal printing process appears to be the optimal solution to predict these errors [222].

As new materials are constantly being developed, simulation programs are also being developed to predict various errors in the production process. This supported the understanding of the additive process; the individual events that operate in the process are essential in achieving the required quality, but especially the efficiency of the process [223]. In terms of importance, the necessary amount of support material in the metal printing process should be kept as low as possible to reduce the time required to construct the part and the needed material and reduce production costs [224,225,226]. At the same time, their correct choice affects not only the mechanical properties of the part but also its microstructure, which depends on the heat dissipation performance dependent on the geometry [227]. The size of the support structure used, the shape, location, and number of contact points, and their orientation should be chosen in the printing process of the part with a certain intention that would provide proper heat dissipation and sufficient strength at the same time [228]. Their correct choice can thus contribute to minimizing the probability of deformation, cracking and overheating of the part [229,230,231]. In the final phase of the process, these support structures are removed manually with pliers and a chisel, by electric discharge machining, or by machining (another need for a fixture), which can increase the overall costs [232,233,234,235,236,237,238]. It is also necessary to consider the achieved surface roughness of the printed parts after the L-PBF process, mainly on curved surfaces, which can lead to anisotropy and affect fatigue life [235,239]. Inappropriate surface roughness, residual stress, and the material’s non-equilibrium phase deteriorate the part’s final properties [240].

Other works presented in the contribution and their detailed description will discuss the possibilities of applying simulation tools in the metal additive manufacturing process, specifically for the L-PBF process. For the area of simulation and prediction of various errors in the process, materials such as aluminum alloys, titanium alloys, copper, stainless steel 316 L, Inconel, or MS 300 type material are the primary materials. In all cases, the main purpose of their study was the application of simulation tools and the ability to predict errors that could occur in the production process. These will be areas such as thermal phenomena in the process, correct orientation of the part, amount of support material, volume fraction, deformation of the part, residual stress, or shape deviation. In all cases, these are factors that can significantly affect the overall functionality of the final part produced by the metal additive manufacturing process. Other works dealt with the results reached by individual authors, as well as their recommendations or observations describing a wide range of possibilities in the field of process analysis of metal additive manufacturing technology using simulation. Effective simulation does not consist of repeated calculations, but of using the right functions that should avoid repeated simulations given what we need to achieve. To start applying simulation tools for the L-PBF process, it is also necessary to consider specific problems associated with their integration at different levels (microscale, mesoscale and macroscale). In his study, Kantaros [241] also pointed to a complex analysis of the integration of simulation systems, their synergistic status, and also the impact on Industry 4.0.

### 2.3. Programs for Simulating the L-PBF Process

#### 2.3.1. Simufact Additive 2022

The Simufact Additive simulation program enables the simulation of metal-based additive manufacturing processes, focusing on laser powder bed fusion (L-PBF) and metal binder jetting (MBJ) processes. It is a software solution designed to predict and compensate for distortion, residual stress, and temperature distribution during printing, heat treatment, cutting, hot isostatic pressing (HIP), and machining processes virtually before a metal 3D printer produces the part. In the case of hot isostatic pressing, bulk powders are densified under the simultaneous effect of high pressure and temperature [242]. The thermal characteristics of the powder bed formed in the metal printing process, such as thermal conductivity or emissivity, need to be considered as another process variable, increasing the complexity of the process [243,244].

The software also allows for exploring a large parameter space, where the simulation identifies the optimal combination of, for example, adjusting the laser power, scanning speed, or temperature settings [245]. Therefore, the software is based on the proven technology of the MARC solver for nonlinear numerical simulations. This allows Simufact Additive to simulate distortion in the part and automatically predefine the component. This ensures high-precision parts from the first print job [246,247].

#### 2.3.2. Ansys Additive

**Figure 10:**
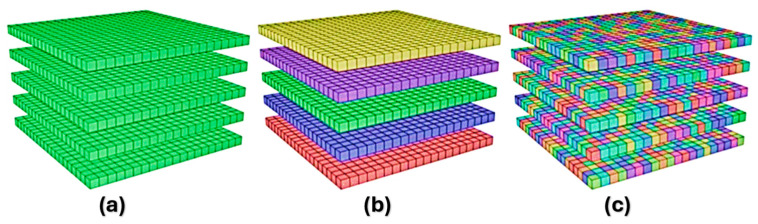
Computational modes of inherent deformation (**a**) uniform stress (**b**) scan pattern (**c**) thermal stress [250].

The program works with voxel or tetrahedral elements, where each finite element layer represents several real layers of metal powder, assuming thermal continuity with the next layer. However, the software does not consider a moving heat source, for example, when the thermal gradient in the direction of construction dominates the thermal gradient in the plane direction due to its influence on residual strains [247,248]. Its advantage is the prediction of layer-by-layer deformation and the peak stress in part, as well as the automatic generation of the load-bearing support, identifying areas with high-stress values [249]. The software uses an inherent method based on the individual stresses arising layer by layer [250]. It offers three levels of simulation—assumed uniform stress, scan pattern, and thermal stress, as shown in Figure 10.

#### 2.3.3. Deform

**Figure 11:**
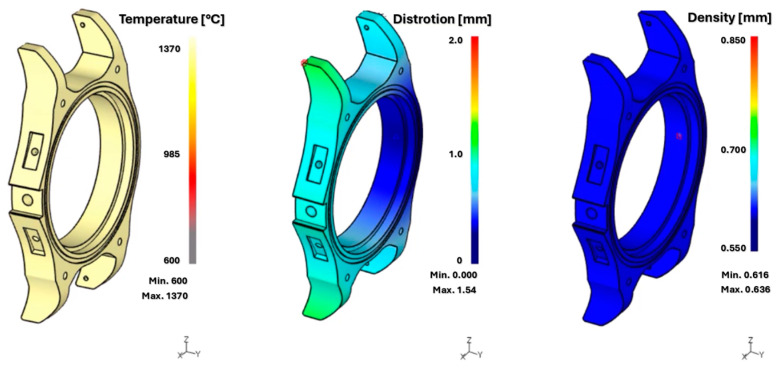
Simulation results in Deform software [251].

Deform software allows the analysis of deformation, densification, mechanical properties, and porosity during the 3D printing phase and after sintering. This reduces times and processes with optimal production performance and a clear improvement in the entire design phase. It allows the identification of errors obtained during sintering and understanding their causes since porosity can affect practically all properties of sintered parts [251,252,253]. At the same time, porosity can manifest itself in the form of irregular pores, e.g., due to insufficient melting, insufficient material supply, which often occurs at the boundary of the molten paths, and shrinkage. The result can be a reduced final strength of the part [254]. The second alternative is the formation of spherical pores resulting from trapped gas, Marangoni turbulence in the melt area, or material evaporation [255,256,257]. Last but not least, the morphology of the powder composition should not be forgotten, which also affects porosity [258,259,260]. Some simulation results are shown in Figure 11.

#### 2.3.4. Amphyon

**Figure 12:**
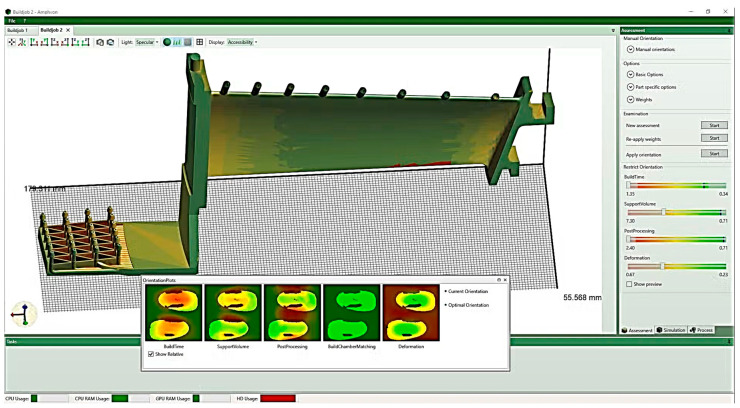
Amphyon simulation software environment.

Amphyon (Figure 12) is a simulation software for powder bed and laser melting additive manufacturing processes involving automated supports. It enables automatic optimization of part orientation, assembly process simulation, and data adaptation to achieve higher quality and stability. It allows users to perform trade-off studies for part orientation depending on accessibility, support volume, assembly time, etc. The deformation calculations from the simulation can be used to compensate for the part geometry to minimize deviation from the design goal [261,262,263,264,265,266].

#### 2.3.5. Netfabb Simulation

**Figure 13:**
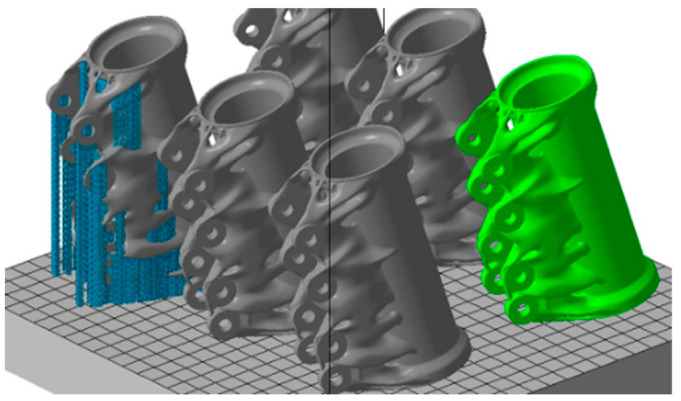
The environment in Netfabb Simulation software.

Using Netfabb Simulation (Figure 13), it is possible to simulate the thermomechanical history of an additive assembly using local simulation focusing on temperature history, deformation, stress and strain, and structural failures. Using customizable supports, thermomechanical simulations can also mitigate excessive distortion during powder bed processing. They also have the advantage of simulating part delamination from the area where structural failure occurred on the component [267,268,269,270,271,272].

#### 2.3.6. VGSTUDIO MAX

**Figure 14:**
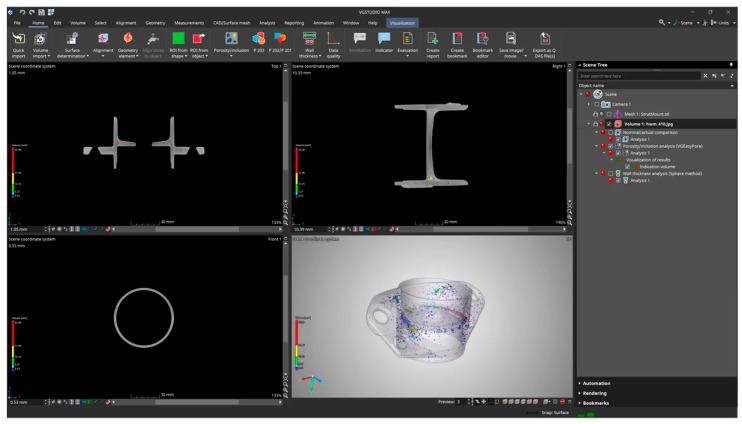
Simulation software VGSTUDIO MAX.

VGSTUDIO MAX software uses a geometry correction module to compensate for deviations and reduce the required design iterations. It also includes a porosity module that allows you to quickly locate any pores, holes, inclusions, or voids within a part in terms of location, compactness, size, and geometry [273,274,275,276,277,278]. A representation of the working environment in the VGSTUDIO MAX simulation program is shown in Figure 14.

#### 2.3.7. AscentAM

**Figure 15:**
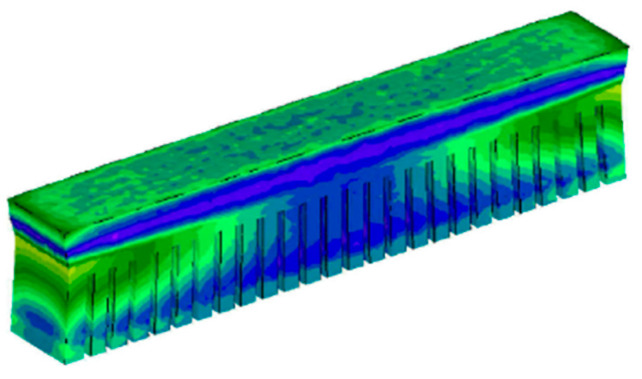
Example of cantilever beam simulation in the AscentAM simulation software [278].

The program (Figure 15) uses the finite element method, with its basic module and several submodules allowing it to simulate thermomechanical processes using a physical approach. Macroscale effects are taken into account to reduce the calculation time. The optimization submodules iteratively pre-deform the geometry of the part based on the structural results using a nonlinear algorithm. Its basic module represents the PBF-LB/M process, which imitates the main manufacturing phases [279,280].

#### 2.3.8. Altair Inspire Print3D 2020

**Figure 16:**
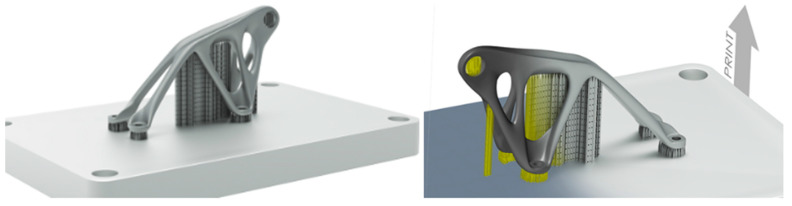
Support generation in Inspire Print3D simulation software [284].

This is a software for part design and simulation of the L-PBF manufacturing process. It allows designers and engineers to use a thermomechanical approach to generate designs and then modify and evaluate them by adjusting process variables. It also has the advantage of generating support structures (Figure 16) as part of the design process, interactively creating them and modifying supports in the same environment as the designed part. It is also possible to evaluate the part layer by layer for overall geometry verification before performing any 3D printing analysis [281,282,283,284].

## 3. Real Use of Simulation Tools for the L-PBF Process

The analysis of the use of simulation tools for the process of metal additive manufacturing was focused on six most common areas:

Section 3.1. Thermal phenomena taking place in the process
Section 3.2. Part orientation and creation of support material
Section 3.3. Volume fraction
Section 3.4. Deformation of the part
Section 3.5. Residual Stress
Section 3.6. Shape deviation

**Figure 17:**
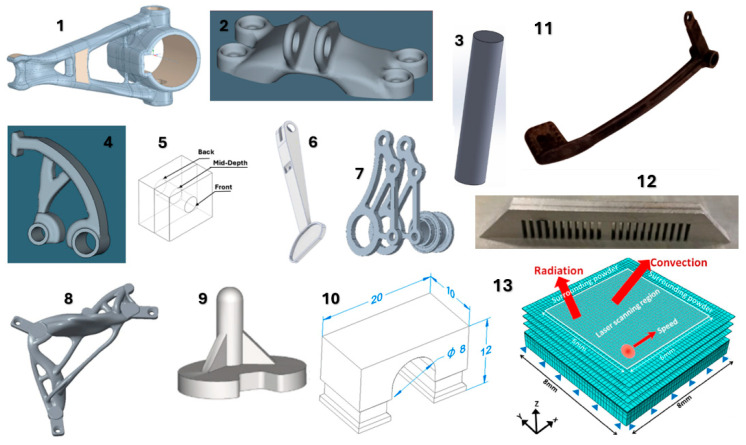
Individual part models, (**1**) slide cylinder model, (**2**) aircraft part, (**3**) tensile test sample, (**4**) part, (**5**) parts with circular inner channels, (**6**) clutch levers, (**7**) rocker arms for racing cars, (**8**) electric motor mounting brackets, (**9**) tibial components, (**10**) bridge-shaped geometry, (**11**) motorcycle brake pedal, (**12**) double cantilever bridge, (**13**) model.

Figure 17 shows the individual models of the parts intended for simulation. Table 1 shows the dimensions of individual CAD models. Color scales complement the simulation results for the above areas.

**Table 1:** Models and dimensions of the parts selected for the simulation.

Sample Marking	Sample Dimension XYZ [mm]	Name of the Part	Reference
Figure 1	110 × 55 × 41	slide cylinder model	[285]
Figure 2	200 × 94 × 62	aircraft part	[185]
Figure 3	diameter 10	tensile test sample	[286]
Figure 4	134 × 162 × 40	part	[287]
Figure 5	10 × 20 × 25	parts with the circular inner channel	[288]
Figure 6	178 × 78.5 × 19.34	clutch lever	[289]
Figure 7	140 × 100 × 85	rocker arm for racing car	[290]
Figure 8	135 × 80 × 65	electric motor mounting bracket	[291]
Figure 9	60 × 40 × 43	tibial component	[292]
Figure 10	10 × 20 × 12	bridge-shaped geometry	[293]
Figure 11	220 × 58 × 50	motorcycle brake pedal	[294]
Figure 12	127 × 12.7 × 18.5	double cantilever bridge	[295]
Figure 13	8 × 8 × 1	model	[296]

### 3.1. Thermal Phenomena Taking Place in the Process

**Figure 18:**
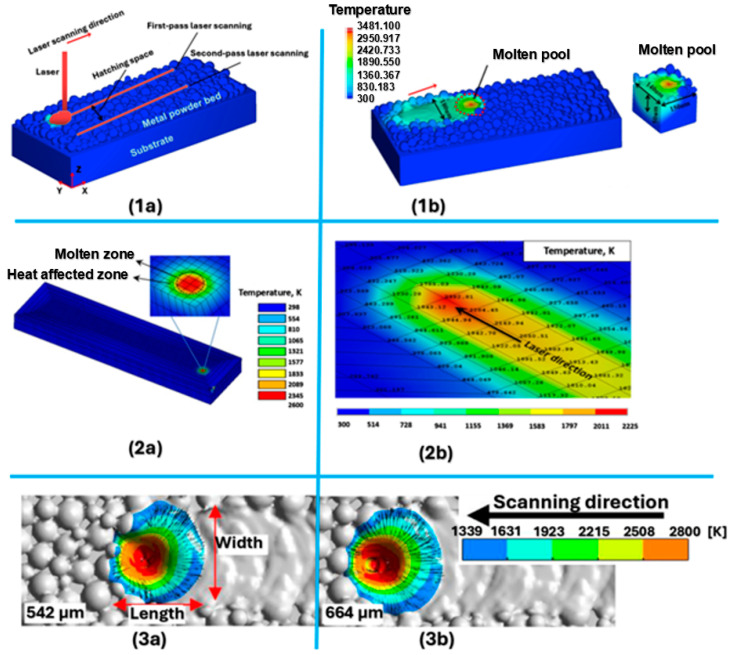
Images of the melting pool in the L-PBF process (**1a**) two-pass model of finite elements under laser action, (**1b**) morphology of the molten pool [297], (**2a**) transient temperature distribution at the beginning of layer melting, (**2b**) temperature distribution at the end of layer melting [298], (**3a**) melt development at different laser deposition rates at 542 μm density, (**3b**) melt development at laser speed 600 mm/s of the powder at 664 μm density [299].

Lu et al. [297] applied mesoscopic scale numerical simulations in their research to predict the flow behavior of a metallic micromolten pool for the L-PBF process. They designed a test that focused on the impact of laser power, hatching space, and laser speed. Hussein et al. [298] created a nonlinear thermo-mechanical 3D FEA model to simulate the temperature and stress fields of finite elements. Due to increasing scanning speeds (200–300 mm/s), there was not enough time for the molten layer to cool down and solidify. The results showed the highest temperature gradients at the beginning of melting the first layer, followed by a decrease at all scanning speeds. On the other hand, Ren et al. [299] found that at a scan speed of 600 mm/s, the melt pool is much more stable at densities of the powder 542 and 664 μs with a near-circular shape. A graphical comparison of the results achieved by the water pool is shown in Figure 18, and the process parameters are described in Table 2.

**Table 2:** Input parameters for L-PBF process simulation and melt pool width and depth comparison.

Reference	Material	Software	Laser Power [W]	Scanning Speed [mm/s]	Layer Thickness [µm]	Hatching Distance [mm]	Molten Area Width [µm]	Molten Area Depth
[297]	AlCu5MnCdVA	EDEM	300	500	50	0.07	140	50
[298]	AISI 316 L	ANSYS	100	300	50	0.075	220–380	400–630
[299]	Cu-Cr-Zr alloy	FLUENT	430	600	61		152.54	139.20

In the first case, Figure 18(1a) was a model showing the final elements of the L-PBF process, mainly containing a laser beam substrate and copper metal powder with a size of 50 μm. In order to realize the study of the overlap rate of neighboring tracks, Lu et al. [297] proposed the use of dual-band laser scanning, where a moving Gaussian surface heat source was chosen as the heat source model. From the results of simulations using the discrete element method (DEM), the geometry of the copper powder layer was obtained, and then the phase transformation process was realized by simulating fluid dynamics. In Figure 18(1b), it was possible to observe a concave shape with a size of 140 μm × 150 μm × 50 μm in the front part of the molten pool. In the upper surface center of the molten pool, the concave depth was small due to the low laser speed of 500 mm/s with little impact on the molten metal. Hussein et al. [298], in the initial phase, focused on the temperature at the beginning of the laser scan, which is shown in Figure 18(2a), where due to the application of a Gaussian heat source, it was possible to clearly see very high temperature gradients located near the laser spot on the powder bed. The cause of this increased temperature of the individual particles of the 316 L powder was the action of the rapidly increasing absorbed energy, as a result of which a molten pool was formed. Exceeding the melting temperature, as well as heat-affected zones around the loose powder, also had an effect on the formation of the molten pool. In the case of the reached temperatures as shown in Figure 18(2b), the highest temperature reached the value corresponding to the melted zone of the powder itself in the form of 2600 K. Nevertheless, this highest temperature at the beginning of the first path and at its end decreased to 2392 K and at the end of the fifth path to 2225 K. This decrease in the maximum temperature was influenced by the increased conductivity of the previously solidified areas of the track, compared to the low thermal conductivity, which was originally available in the powder bed itself.

Among other things, it was necessary to think about the fact that the thermal field changed due to the movement of the laser along the track, where the melt pool moved simultaneously with the laser source. Ren et al. [299] confirmed the above statement based on a similar analysis which they performed at a speed of 1200 mm/s. They achieved a significantly elongated shape of the melting pool, where the molten area width reached a value of 100.32 μm and the molten area depth reached 165.89 μm. The energy obtained by the local spot with a scan speed of 1200 mm/s was significantly reduced, resulting in less melt flow. This resulted in a narrow width of the melt pool. Through the analyses, it was possible to confirm that the drop in effective laser absorption at 1200 mm/s can be attributed to the fact that the melt bath had a shallow penetration into the substrate. On the contrary, compared to the scanning speed of 600 mm/s, as shown in Figure 18(3a), a deeper penetration was achieved, which was able to increase the laser absorption. Among other things, the influence of the preheating effect at the high speed of 1200 mm/s was less compared to the low speed of 600 mm/s, resulting in worse melt spreading. This poor melt spreading ultimately led to melt pool instability in the form of an irregular melt pool shape as well as distortion of the track morphology.

After the metal printing process, the parts are additionally annealed, which relieves the residual stress, which can optimize the mechanical properties of the parts (strength, resistance, and fatigue resistance) [300,301].

### 3.2. Build Orientation

**Figure 19:**
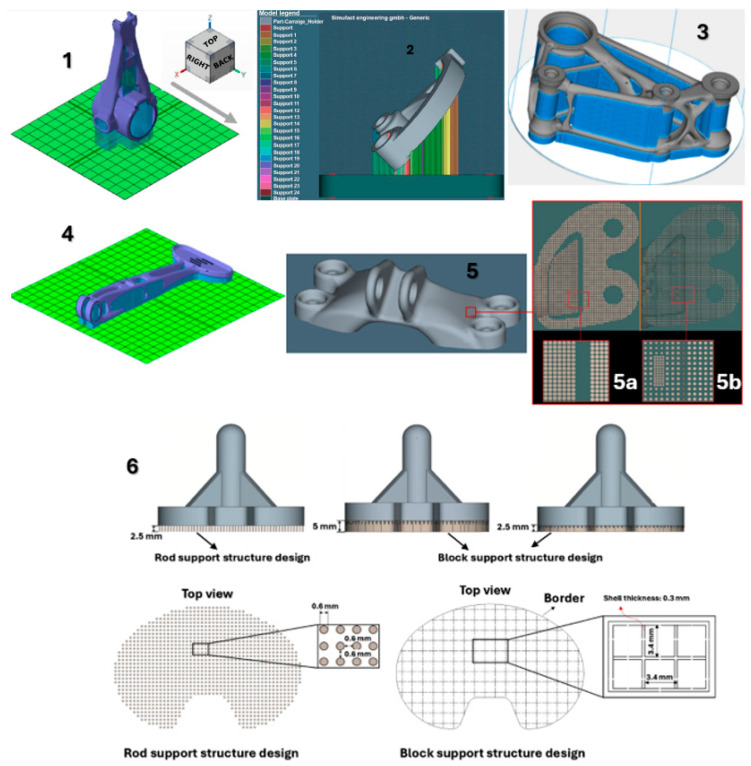
Comparison of the display of the orientation of the part and the support material (**1**) model of the sliding cylinder [285], (**2**) part [287], (**3**) rocker arm for a racing car [290], (**4**) clutch lever [289], (**5a**) layout of the support structure of the aircraft part without optimization, (**5b**) with optimization [185], (**6**) tibial component [292].

The orientation of the part during the production process is influenced, in addition to the number of individual layers used, by the choice of the geometry of the carrier material, which in the final phase also affects the distribution of residual stresses. The orientation of the part affects the resulting mechanical properties of the part and the production time. [302,303]. Pagac et al. [285] analyzed as many as nine possible iterations, based on which they obtained the optimal orientation of the part for the process of its construction (see Figure 19(1)). It took into account parameters such as the volume of the carrier material, the surface area of the carrier material, the height of the model’s position, and the height of the model’s center of gravity. Similarly, Kaščák et al. [287] analyzed the suitable orientation of the part (Figure 19(2)) for the construction process by applying a simulation tool, which took into account the so-called weighting factor in the form of criteria (support area, support size, and building risk) for the analysis of the simulation. Markovits et al. [290], by predicting the appropriate orientation of the weight component in the Racing Car Chassis suspension section using simulation, reduced the weight by up to 40% per component and achieved a 3x higher load capacity. The result was the orientation of the part to Figure 19(3), where the weight of the support was 67 g, and the printing time of the part construction was 10.1 h. Mikulikova et al. [289] achieved the best part orientation for manufacturing when producing a clutch lever for a sports car, as shown in Figure 19(4). This allowed them to achieve a lower volume of carrier material, which significantly impacted heat dissipation and deformation of the part in the printing process. The process parameters designed to simulate the part’s orientation are described in Table 3, and their results concerning the shape of the part are shown in Figure 19.

**Table 3:** Process parameters designed to simulate the orientation of a part.

References	Software	Material	Laser Power [W]	Scanning Speed [mm/s]	Layer Thickness [µm]	Hatching Distance [mm]	Element Size [mm]	Results [mm^3^]
[285]	Autodesk Netfabb	AISI 316 L	200	650	50	0.11	1	28.888
[287]	Simufact Additive 2022	AlSi10Mg	200	800	30	0.08	2	14.946
[290]	ANSYS 2020R1	Ti6Al4V	200	600	20	0.1	1	10.1 g
[289]	Simufact Additive	AlSi10Mg	195	800	30	0.09	1	12.586
[185]	Simufact Additive 2022	AlSi10Mg	200	800	30	0.08	2	42.693
[292]	Simufact Additive 2020	Ti-6Al-4V	180	1250	30	0.105	0.5–1.5	1560

The prediction of the appropriate orientation of the part concerning the geometry of the generated support structures without the optimization of the support—Figure 19(5a)—and the use of the function of optimization of the support generation—Figure 19(5b)—was dealt with by Kaščák et al. [185]. Cylindrical shape geometry was used for both support formation cases. The simulation showed a change in the representation of the generated support structures’ density and the created support’s spacing and size (as shown in Figure 19(5a,5b)). The support volume without optimization mode reached 71,875.6 mm^3^; with support optimization, it reached 42,693.7 mm^3^ [185]. For a similar analysis, Ninpetch et al. [292] applied a simulation tool that focused on the design of the tibial component support structures, using two types of support structures with a height of 2.5 mm and 5 mm—Figure 19(6). They found that reducing the height of the support lowers both distortion and material consumption and that a block structure instead of a bar structure provides a more effective reduction in deformation than the reduced height of the support. The support volume for constructing the supporting structure with a case height of 5 mm and a case height of 2.5 mm was 1560 mm^3^ and 780 mm^3^ [292].

### 3.3. Volume Fraction

In the additive process, the total energy associated with the production of a component is defined as its volume fraction. The individual calculation modules in the simulation tools make it possible to analyze the microstructure represented by phase fractions, which are calculated by solving diffusion-type equations controlled by temperature changes. As part of computational analyses, simulation programs work with voxel elements containing hexahedral elements for the so-called discrete representation of the part, which works in combination with the volume fraction of the element.

A representative volume element usually contains many crystals, so the geometry of the microstructure is not distinguished. These models are less accurate than micro and mesoscale models but are more computationally feasible in a fully integrated modeling framework. By controlling such a volume fraction in the process of metal additive manufacturing, it is possible to monitor the course of the microstructure in the geometry of the part. At the same time, it is possible to use the so-called process maps to define the scope of its control. Likewise, the analysis of the volume fraction of a part provides us with flexibility, which is invaluable in product development. It allows one to generate and locally control the part geometry structure and the material’s behavior in each volume element—voxel in part [304].

**Figure 20:**
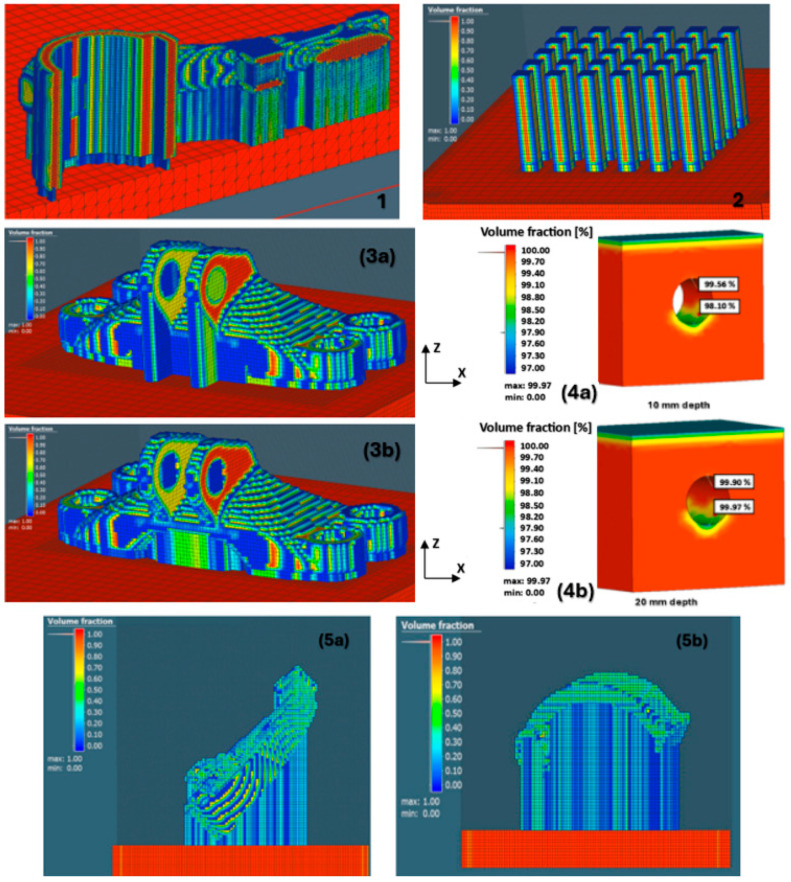
Comparison of the display of the volume fraction of the material under different input conditions and for different types of parts in the L-PBF process (**1**) model of the feed cylinder [285], (**2**) tensile sample [286], (**3a**) volume fraction of the material of the aircraft part without optimization, (**3b**) with optimization [185], (**4a**) part with an internal circular channel with a wall thickness of 10 mm, (**4b**) part with an internal circular channel with a wall thickness of 20 mm [288], (**5a**) volume fraction in case of appropriate part orientation, (**5b**) volume fraction in case of inappropriate part orientation [287].

Its additional advantage is controlling the geometry structure in each volume element (voxel) component [305]. This affects not only the production of the part but also the design, optimization, and control of the proposed materials. This value describes how much of the volume of the voxel element fills the part’s geometry. If the volume fraction value of 1 is reached, the voxel element is 100% inside the part geometry. The voxels on the part’s surface should show very low volume fractions. The display of the results of the inspection of the volume fraction of the part concerning the shape of the part is shown in Figure 20, where the specific process conditions are shown in Table 4.

**Table 4:** Process parameters designed for the simulation of the volume fraction of a part.

References	Software	Material	Laser Power [W]	Scanning Speed [mm/s]	Layer Thickness [µm]	Hatching Distance [mm]	Element Size [mm]
[285]	ANSYS 2020R1	AISI 316 L	200	650	50	0.11	1
[286]	Simufact Additive 2020	AISI 316 L	195	800	20	0.09	1
[185]	Simufact Additive 2022	AlSi10Mg	200	500	30	0.07	2
[288]	Simufact Additive 2023	MS 300	200	350	30	0.12	1
[287]	Simufact Additive 2022	AlSi10Mg	200	800	30	0.08	2

In multiple research results [185,284,285,286], the color representation of the given factor was monitored, which can be used to determine the quality of the network. For all of these cases, a suitable network quality was achieved. Kaščák et al. [185] used the simulation tool to evaluate the volume share concerning the conventional method of generating support and the function enabling support generation using the optimization mode. The simulation results showed, with the help of the support optimization function, a reduction in the volume fraction of the part, as shown in Figure 20(3a,3b).

Silva et al. [288] simulated the volume fraction factor concerning the phase transformation rate KM 0.033 at two different material thicknesses, namely 10 mm and 20 mm, with an inner hole with a diameter of 8 mm—Figure 20(4a,4b). In terms of the phase volume fraction of the simulated parts, a predominantly martensitic microstructure with occasional austenite-containing regions was achieved. The impact of phase transformation was only minimal.

### 3.4. Distortion

**Figure 21:**
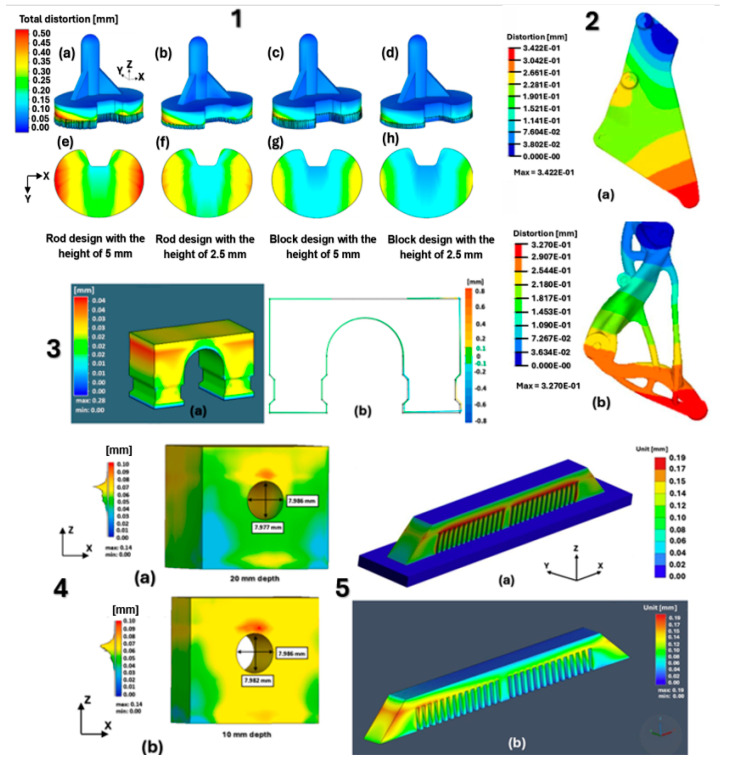
The comparison of the deformation display under different input conditions and for different types of parts in the L-PBF process (**1**) tibial component model [292], (**2**) electric motor mounting bracket [291], (**3a**) comparison of simulated bridge-shaped geometry (**3b**) geometry of the original model [293], (**4a**) part with an internal circular channel with a wall thickness of 10 mm, (**4b**) part with an internal circular channel with a wall thickness of 20 mm [288], (**5a**) Simulated deformation field for a double cantilever beam before cutting off the supports by the self-strain method (**5b**) by the simulation method [295].

Distortion is a very important factor that can harm the geometric and dimensional accuracy of the final manufactured parts in the L-PBF process in metal additive manufacturing. The resulting thermal gradients in the production process contribute to a large thermal stress, which is ultimately reflected in the deformations of the manufactured part. This thermal stress can also arise due to uneven expansion or contraction of the molten and solidified layers. The material exposed to this heat-affected zone undergoes thermal expansion, causing the part to bend downwards while the top layer remains hot. On the contrary, the cooling of the material is manifested by thermal contraction in the upper part of the part. One of the ways to prevent this plastic deformation is to preheat the build platform before production, constantly heating the build platform or the structural chamber during the production process. The result of preheating or constant heating is a reduction in deformation and therefore a reduction in temperature gradients. The process parameters specified for the deformation simulation are described in Table 5, and the distortion results concerning the shape of the part are shown in Figure 21.

**Table 5:** Process parameters designed for deformation simulation.

References	Software	Material	Laser Power [W]	Scanning Speed [mm/s]	Layer Thickness [µm]	Hatching Distance [mm]	Element Size [mm]	Results [mm]
[292]	Simufact Additive 2020	Ti-6Al-4V	180	1250	30	0.105	0.5–1.5 (1)	0 ÷ 0.05
[291]	Simufact Additive	350	350	1150	50	0.17	2.12 (2)	36%
[293]	Simufact Additive 2020	AISI 316 L	400	-	50	-	(3)	0.04
[288]	Simufact Additive 2023	MS 300	200	350	30	0.12	1 (4)	0 ÷ 0.1
[295]	Simufact Additive 3.1	Inconel 718	350	1150	50	0.17	2.12	0 ÷ 0.19

By using simulation as shown in Figure 21(1), Ninpetch et al. [292] clarified that reducing the height of the support reduces the distortion of the tibial component and material consumption. However, removing the building part and support is more difficult. Near the interface between the tibial tray and the supporting structure, there was more distortion due to the different stiffness of the solid volume and the supporting structure. The maximum distortion occurred around both edges of the long side of the tibial tray. Overall distortion distribution of the workpiece with different support structures and support heights. Figure 21(1a,1e) 5 mm high bar support structure (S-T8); Figure 21(1b,1f) 2.5 mm high bar support structure; Figure 21(1c,1g) 5 mm high block support structure; Figure 21(1d,1h) 2.5 mm high block support structure. Bassoli et al. [291] redesigned the aluminum component by optimizing topology and design for additive manufacturing. The results showed that the optimized console guaranteed a 36% reduction in maximum offset as shown in Figure 21(2). FEM results for displacements and Von Mises stresses for the original (Figure 21(2a)) and redesigned cantilever after topological optimization (Figure 21(2b)). Cantilever geometries are typically used in the study of residual stresses and deformations in the L-PBF process due to their simple but significant overhang characteristics [248,306].

Similar research on the analysis of distortion or deformation was carried out by Hajnys et al. [293], who compared the results of the simulation of the distortion of a part with a real part whose geometry corresponded to the shape of the bridge—Figure 21(3). They did not use any supporting material. Silva et al. [288] considered the effect of the phase transformation rate of KM 0.033 at two different material thicknesses of 10 mm and 20 mm with an internal hole with a diameter of 8 mm on the deformation results, see Figure 21(4). The results showed uniform deformation for the stated phase transformation rate, achieving a higher level at the channel’s top. The transition of the red color was conditioned by the deflection mechanism, which allowed deformation in the direction of the axis of Z. Chen et al. [295] applied a simulation tool to compare the distortion profile of a double cantilever beam, see Figure 21(5a,5b), calculated by the inherent strain method with the profile obtained by simulation, and the results were similar. The overall distortion on the top surface was small due to the right and left side beams still fixed on the support columns. A large deflection was observed along the joints between the beam and the support columns in the inherent stress method and the Simufact simulation mode.

### 3.5. Equivalent Stress

As a result of the action of thermophysical phenomena, thermal residual stresses are generated in the L-PBF process, which have to be predicted. It is also necessary to predict parameters such as the temperature field of the molten pool, microstructure, and deformations [304,305,307,308]. In the case of residual stress, this is an important factor affecting the structural properties of the part [296,309,310]. Its main source is primarily the thermal cycle, where the laser scans every single layer during the production process, where the previously solidified layers are melted and cooled several times at inconsistent heat levels.

**Figure 22:**
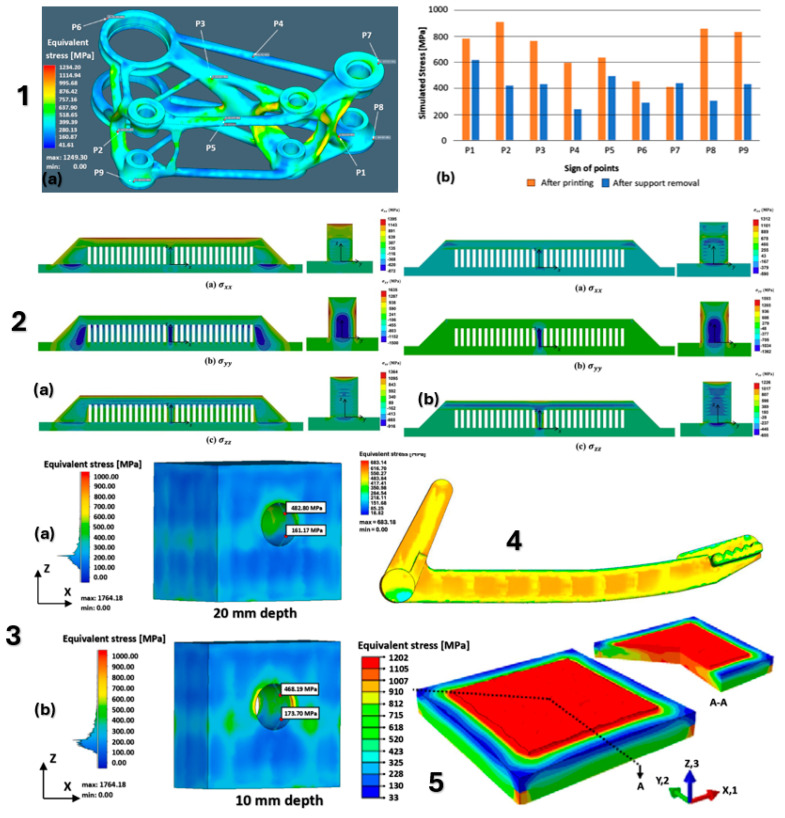
Comparison of the equivalent stress display under different input conditions and for different types of parts in the L-PBF process (**1**) rocker arm for a racing car [290], (**2a**) double cantilever beam before removing the support, (**2b**) after removing the support [295], (**3a**) part with an internal circular channel with a wall thickness of 10 mm, (**3b**) part with an internal circular channel with a wall thickness of 20 mm [288], (**4**)—motorcycle brake pedal [294], (**5**)—model [296].

In connection with the gradient of stresses in one layer of the part, the two most important areas are formed during heating: the upper part of the layer exposed to the laser and the interface between the layer and the previous layer [311]. In general, it is given that residual stress magnitudes are higher in the longitudinal direction than in the transverse direction due to a higher temperature gradient along the direction of construction of the part [312]. As a result of their occurrence, cracking [313], delamination [314], or deformation of the part can occur if their sizes exceed the yield strength or tensile strength [315,316]. Process parameters designed for the simulation of equivalent stresses are listed in Table 6, and their results for the shape of the part are shown in Figure 22.

**Table 6:** Process parameters designed for simulation of equivalent stress.

References	Software	Material	Laser Power [W]	Scanning Speed [mm/s]	Layer Thickness [µm]	Hatching Distance [mm]	Element Size [mm]	Results [MPa]
[290]	ANSYS 2020R1	Ti6Al4V	200	600	20	0.1	1	200 ÷ 600
[295]	Simufact Additive 3.1	Inconel 718	350	1150	50	0.17	2.12	1400 ÷ 1600
[288]	Simufact Additive 2023	MS 300	200	350	30	0.12	1	470 ÷ 620
[294]	Simufact Additive 2021	AISI 316 L	200	650	50	0.11	2	362 ÷ 504
[296]	ABAQUS	In718	600	1000	30	0.4	0.2 × 0.2 × 0.015 mm	1202

Markovits et al. [290] used the simulation program Simufact Additive, which is capable of assessing the load of the part in the form of an estimate of stresses and their distribution during various operating steps. The variety of individual points corresponded to the entire part, which led to the conclusion that in the post-print state, the stress values were below the yield strength of the material. The residual stresses in the part were 200 to 600 MPa, as shown in Figure 22(1). Figure 22(1a) shows the point locations for comparing simulated print stress results. These are the stress values simulated at each point in different states. The points were randomly selected for comparison. The simulated stress values of nine selected points (P1–P9) for different process steps are shown in Figure 22(1b). The nine selected points are located at different locations in the model, representing the entire part. It can be seen from the simulated stress values that in the elastic range, values below the yield strength of the material are generated in the as-printed state. Chen et al. [295] calculated the distribution of residual stresses for a double cantilever beam in the actual state (before the removal of the support), where the tensile stress in the applied upper layer was caused by the contraction of the molten material after cooling. Both longitudinal and transverse tensile stresses were close to surfaces, and internal compressive stresses reached values of 1400 MPa and 1600 MPa. Therefore, the yield strength of Inconel 718 was exceeded. In contrast, removing the supports significantly relieved the normal residual stresses in all directions except the central support, as shown in Figure 22(2a,2b). Silva et al. [288] achieved significantly higher values of equivalent stresses on the inner surface of the duct due to the phase transformation rate KM 0.033 at two different material thicknesses of 10 mm and 20 mm with an inner hole of 8 mm, caused by changes in heat dissipation mechanisms, as shown in Figure 22(3a,3b). Fojtík et al. [294] achieved the highest residual stress values on the outer surface of the part. A gradual decrease in the magnitude of the stress from the surface towards the center of the part could be seen, as shown in Figure 22(4).

Cheng et al. [296] analyzed the residual stresses after cooling the part to a room temperature of 20 °C at a time t = 2000 s. The maximum stress was reached on the upper area of the applied layers with a value of 1202 MPa. Plastic deformation of the material occurred due to exceeding the material yield strength. Conversely, low stresses could be observed along the edges in the upper region of the model, where it increased towards the bottom surface, as shown in Figure 22(5).

### 3.6. Shape Deviation

**Figure 23:**
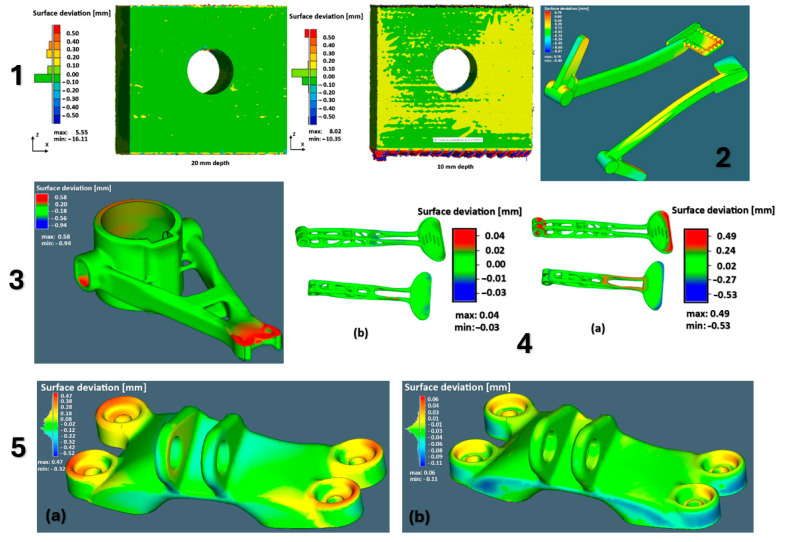
Comparison of the display of the shape deviation under different input conditions and for various types of parts in the L-PBF process (**1**). part with an internal circular channel with a wall thickness of 20 and 10 mm [288], (**2**). motorcycle brake pedal [320], (**3**). slider cylinder model [285], (**4**). clutch lever [289], (**5**). aircraft part (**5a**) aircraft part shape deviation without optimization, (**5b**) with optimization [185].

The cause of shape deviations during the L-PBF process is the occurrence of large thermal gradients, which lead to high thermal stress [317]. At the same time, these shape deviations are due to the influence of uneven expansion and the contraction of molten and solidified layers during the process [318]. These shape deviations can be mitigated by correctly selecting input parameters for the L-PBF process, such as scan speed, laser power, scanning strategy, and layer thickness [319]. Process parameters designed to simulate shape deviations are listed in Table 7, and their results for the shape of the part are shown in Figure 23.

The research carried out by Silva et al. [288] focused on the application of a simulation of the shape deviation concerning the phase transformation rate of KM 0.033 at two different material thicknesses, namely 10 mm and 20 mm with an inner hole of 8 mm. The color gradient of the 20 mm geometry exhibited a more regular behavior because it presented essentially only one color, as shown in Figure 23(1). Thus, quantitatively, the scale of surface deviation of the geometry of 20 mm was more accurate, with the error being predominantly between −0.1 and 0.0 mm, while in the 10 mm geometry, the error was between −0.1 and 0.1 mm in the surface and channel of the geometry.

Results by Kudrna et al. [320] showed an upward bend compared to the original shape at both ends of the part, which indicated a slight distortion of the lower surface of the bar part. The overall deformation of the brake pedal was thus slightly tilted. As part of the results, the maximum distortion was 0.76 mm at the end of the pedal and the minimum distortion was −0.81 mm at the end of the rod (cylinder), as shown in Figure 23(2).

A simulation tool for predicting distortion was used in the research by Pagac et al. [285]. The total displacement in the range of 0.6–0.7 mm was the result of the simulation with the base plate. The deviation of the surface deformation was 0.58 mm, with the largest deformation evaluated on the nose of the component, as shown in Figure 23(3). Mikulíková et al. [289] applied a simulation program to the part geometry distortion area, and their results demonstrated a maximum deviation of 0.49 mm and a minimum deviation of −0.53 mm, while not using the compensation function, as shown in Figure 23(4a). In contrast, when the compensation was used, maximum deviations with a value of 0.04 mm and a minimum value of −0.03 mm were obtained, as seen in Figure 23(4b).

**Table 7:** Process parameters designed to simulate shape deviation.

References	Software	Material	Laser Power [W]	Scanning Speed [mm/s]	Layer Thickness [µm]	Hatching Distance [mm]	Element Size [mm]	Results [mm]
[288]	Simufact Additive 2023	MS 300	200	350	30	0.12	1	−0.1 ÷ 0.1
[320]	Simufact Additive 2020	AISI 316 L	200	650	50	0.11	2	0.76
[285]	Autodesk Netfabb	AISI 316 L	200	650	50	0.11	1	0.6 ÷ 0.7
[289]	Simufact Additive	AlSi10Mg	195	800	30	0.09	1	−0.03 ÷ 0.04
[185]	Simufact Additive 2022	AlSi10Mg	200	800	30	0.08	2	−0.11 ÷ 0.06

Kaščák et al. [185] analyzed the evaluation of shape deviations using a simulation tool for the conventional method of generating support and the function allowing support generation using optimization mode. Without using the support optimization function, surface shape deviations were achieved in the range from min. −0.52 mm to max. 0.47 mm. When using the support optimization function, surface shape deviations achieved ranged from min. −0.11 mm to max 0.06 mm, as shown in Figure 23(5a,5b).

## 4. Discussion

The high print failure rate in metal additive manufacturing threatens not only its economic feasibility but also thwarts efforts to use it in high-volume production. One of the ways to eliminate this is the use of simulation tools, which aim to provide engineers and designers with the means to analyze the behavior of the part in various design conditions and provide the insight necessary to make “first correct prints” a reality. These simulation tools provide an environment in which engineers can model and simulate multiple physical aspects, such as heat transfer and phase changes, as well as track the evolution of material properties throughout a build. In other words, the software provides multi-scale, multi-physics capabilities that support the complex interaction of multiple algorithms and processes.

By applying simulation tools to the process, it is possible not only to avoid a larger number of iterations, but also to comprehensively assess the impact of any process parameter on the overall quality of the part and thus achieve appropriate price efficiency. With the development of new materials and a more compact geometry of the part, considering the final properties that are a condition for its functionality for a specific application, it is necessary to constantly know and control the metal additive manufacturing process. These simulation tools are the way to achieve this and be sure that the correlation of all parameters that interact with each other during the process will create a fully functional part considering the required properties.

Numerous papers currently focus on using simulation to determine the appropriate part orientation, displacement, deformation, etc.; however, very few studies compare the obtained deviations of shape and dimensional accuracy using the digitization method. Therefore, future studies should focus on these areas. Similarly, a simulation could focus on microstructure resulting from the way the atoms are arranged in the metal, which determines the physical and mechanical properties of the material.

At the same time, it would be possible to compare the results of the simulation regarding the microstructure with monitoring the morphology of the melt layer through electro-optical control. Other methods that could be linked to the simulation would be porosity monitoring using ultrasonic testing technology, as the changes that occur during laser performance determine the formation of a porous layer.

Another possibility that has its justification is the monitoring of the fusion of the L-PBF process itself, the monitoring of which can give all the information not only about the stability of the process but also about its errors. This would involve the integration of simulation tools in connection with process monitoring. This would improve the accuracy of the results. Among other things, the question dealing with active online monitoring has its weight in the future, as the conventional way of monitoring the L-PBF process in the case of optical imaging may have some limitations, such as lighting, temperature, or evaporation of metals. The effectiveness of online monitoring is the active emission of rays, reducing not only the requirements for the working environment, but also improving the sensitivity of the measurement. Closely related to this is the possibility of real-time interactive data, where a large amount of information can be obtained because of monitoring, which is based on various machine algorithms. Such data monitoring or real-time monitoring systems could provide real feedback during the monitoring process, which in the final stage will not only improve the efficiency of the control process, but also its accuracy.

An area of future research could also focus on the post-processing of additively manufactured metal parts to improve surface integrity, including surface roughness, subsurface residual stresses, and microstructure. It mainly concerns the investigation of the effect of residual stress on the machinability properties of metal parts. This is a broad area, given the number of factors that enter the machining process, such as tool wear, cutting forces, etc. The effect of preheating the build platform, which can have a positive effect on the thermal properties of the part, could also be investigated, and the research could also be expanded to include the effect on the change in microstructure (size, density and orientation of grains).

A closer comparison of the features and capabilities of the simulation tools discussed in the paper is described in Table 8.

**Table 8:** Comparison of the features and capabilities of the simulation tools discussed.

Software Features	Edem	Ansys Additive 2020R1	Fluent	Netfabb	Simufact Additive 2022	Amphyon	Abaqus
Import support	no	no	no	yes	yes	yes	no
Porosity	yes	no	yes	no	no	no	no
Microstructure evaluation	yes	no	yes	no	not included in this version	no	yes
Displacement	no	yes	yes	yes	yes	yes	yes
Building job simulation	no	yes	yes	yes	yes	yes	yes
Stress	no	yes	yes	yes	yes	yes	yes
Heating treatment simulation	yes	no	yes	yes	yes	yes	yes
Orientation suggestion	no	no	no	no	yes	yes	yes
Input file format	-	.stl	-	.stl	.stl	.stl, obj	-
Output file format	-	.avz, VTK, .stl, CSV	-	.stl, CLI	.stl	.stl, CLI	-
Recoater crash	no	yes	no	yes	yes	yes	no
HIP	no	no	no	no	yes	no	yes
Shrinkage prediction	no	yes	yes	yes	yes	yes	yes
Hot spot	yes	yes	yes	yes	no	no	no
Roughness prediction	no	no	no	no	yes	no	no
Displacement in support	no	yes	yes	yes	yes	yes	yes
Estimated print time	no	no	yes	yes	yes	no	yes
Defect distribution	yes	no	yes	no	yes	no	yes

## 5. Conclusions

This paper presented a comprehensive overview of the application of simulation tools based on the opportunities and applications of current technologies in metal additive manufacturing. It highlighted the importance of modeling and optimizing production processes in a real environment. The paper also focuses on the significance of various process parameters set in the simulation environment and their mutual correlation, as well as the obtained results and their verification in the real production of the part.

Based on the research presented, the following conclusions were formulated:

- Simulation tools can identify adverse phenomena occurring in the production processes.
- They reflect the functioning of systems in the production environment, which are subjected to various analyses.
- With their help, it is possible to test the validity of the proposed conceptual and model solutions without making actual changes in the production system, which would incur significant expenses.
- Simulation tools have evolved to have a measurable impact on the design and production of quality parts. However, in the case of design, it is not only about the traditional product design, i.e., the geometry of the part but also about the design of the parameters of the machine structure, the orientation of the part, the choice of the geometry of the support formation, and the steps of subsequent processing.
